# Lyme Disease Frontiers: Reconciling *Borrelia* Biology and Clinical Conundrums

**DOI:** 10.3390/pathogens8040299

**Published:** 2019-12-16

**Authors:** Vladimir V. Bamm, Jordan T. Ko, Iain L. Mainprize, Victoria P. Sanderson, Melanie K. B. Wills

**Affiliations:** G. Magnotta Lyme Disease Research Lab, Molecular and Cellular Biology, University of Guelph, 50 Stone Road East, Guelph, ON N1G 2W1, Canada; vbamm@uoguelph.ca (V.V.B.); koj@uoguelph.ca (J.T.K.); imainpri@uoguelph.ca (I.L.M.); vsande01@uoguelph.ca (V.P.S.)

**Keywords:** Lyme disease, *Borrelia*, pathogenesis, virulence, predisposition, antimicrobials, pleomorphy, serotype, chronic Lyme disease, post-treatment Lyme disease syndrome

## Abstract

Lyme disease is a complex tick-borne zoonosis that poses an escalating public health threat in several parts of the world, despite sophisticated healthcare infrastructure and decades of effort to address the problem. Concepts like the true burden of the illness, from incidence rates to longstanding consequences of infection, and optimal case management, also remain shrouded in controversy. At the heart of this multidisciplinary issue are the causative spirochetal pathogens belonging to the *Borrelia* Lyme complex. Their unusual physiology and versatile lifestyle have challenged microbiologists, and may also hold the key to unlocking mysteries of the disease. The goal of this review is therefore to integrate established and emerging concepts of *Borrelia* biology and pathogenesis, and position them in the broader context of biomedical research and clinical practice. We begin by considering the conventions around diagnosing and characterizing Lyme disease that have served as a conceptual framework for the discipline. We then explore virulence from the perspective of both host (genetic and environmental predispositions) and pathogen (serotypes, dissemination, and immune modulation), as well as considering antimicrobial strategies (lab methodology, resistance, persistence, and clinical application), and borrelial adaptations of hypothesized medical significance (phenotypic plasticity or pleomorphy).

## 1. Introduction

Evidence of the illness that would come to be called Lyme disease (or Lyme borreliosis) began accumulating in Europe in the late 19th century [1]. However, it was not until the 1980s that a causative pathogen, *Borrelia burgdorferi,* was discovered and characterized as a result of public health investigations into a cluster of mysterious disease cases on the American Eastern seaboard [2,3]. In the ensuing decades, much has been learned about the spirochetal pathogen and the dynamic host interplay that ultimately gives rise to Lyme disease (LD). Yet, incidence has continued to climb in the United States, where it is estimated that over 300,000 new cases occur every year [4,5], and concerns persist about diagnostic testing, treatment, and longstanding complications. Indeed, as the most prevalent vector-borne disease in the Northern Hemisphere [6,7], LD is increasingly recognized as an escalating public health threat that demands innovative strategies for prevention and care. Efforts to manage this modern epidemic are necessarily multidisciplinary [8], considering the complexity of the pathogen, the enzootic cycle that maintains it in nature, and the highly variable human disease that arises from it.

The original *Borrelia* genus contains two groups of medical significance, one encompassing the organisms responsible for relapsing fever, and the other now widely referenced as the Lyme complex (formerly *Borrelia burgdorferi* sensu lato, or s.l.) [9]. To reflect the genomic distinctions between the two groups, a proposal was recently made to divide the genus and capture Lyme pathogens in a new designation, “*Borreliella*” [10], which has not been universally endorsed [11]. For the purposes of this review, Lyme complex, Lyme Borreliosis group, *Borrelia*, *B. burgdorferi* s.l., Bb, and *Borreliella* should be considered synonymous and indicative of the Lyme-disease (borreliosis) causing spirochetes. 

The genome of Lyme spirochetes has been described as the most complex of all bacteria, owing to its linear chromosome supplemented with more than 20 linear and circular plasmids, of which several encode essential proteins [12,13]. At 1.5 Mb, it is not, however, the largest microbial genome on record, and in fact *Borrelia* relies on its hosts to fulfil basic biosynthetic functions because it lacks fundamental machinery for biogenesis [14]. 

The continuity of *Borrelia* in the wild is due to its persistent colonization of reservoir species like the white-footed mouse (*Peromyscus leucopus*). Vector-competent ticks, such as *I. scapularis*, and *I. pacificus* in North America, and *I. ricinus* in Europe, acquire the pathogen from the reservoir during a blood meal, and can then transmit the spirochete to a new host during a subsequent feed [15]. Although Lyme is often considered a disease acquired in nature, compelling investigations suggest that a high proportion of human tick encounters occur in residential areas [16], and although the tick density in urban green spaces is generally lower than in natural forests, the prevalence of *Borrelia* can be higher [17]. Adventitious ticks introduced by migratory birds, for example, may also establish new populations [18,19,20]. 

In humans, tick-transmitted infection begins at the bite site, which may be demarcated by an erythema migrans (EM) or bull’s eye rash, and may also be accompanied by flu-like symptoms. As the spirochetes migrate away from the lesion via vasculature and lymphatics, they can invade distal sites and manifest in the skin, joints, heart, nervous system, endocrine glands, and gastrointestinal tract [21]. This “great imitator” can cause debilitating illness that mimics conditions such as multiple sclerosis and cancer [22]. The presentation of LD can vary considerably between individual patients, and also between cohorts from different geographic regions. For instance, European LD is characterized by skin disorders, such as acrodermatitis chronica atrophicans and borrelial lymphocytoma, which are atypical for North American LD [23]. The distinct distribution of genospecies and serotypes in North America versus Europe, for example, appears to be a major determinant of the intercontinental variability in the prevalence of specific symptoms [24]. Prompt treatment with antibiotics during the acute phase of the infection predicts the best outcome, although even gold-standard care does not guarantee complete resolution of symptoms and functional impairment [25]. Delays in diagnosing and treating the disease have been associated with worse prognoses [26].

**Figure 1:**
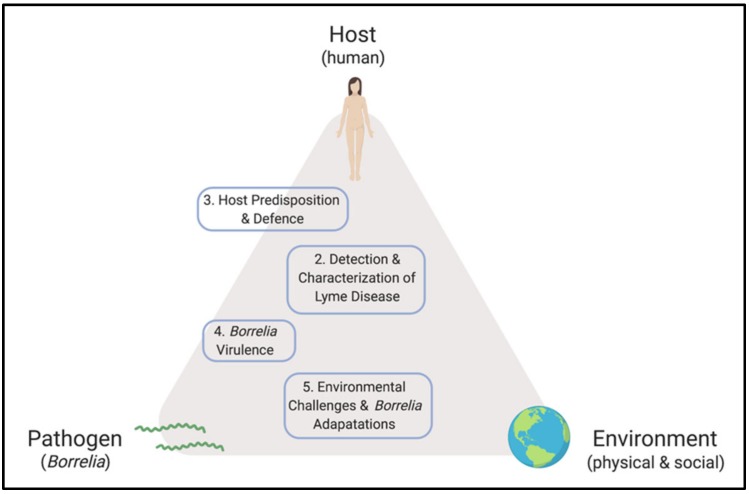
Concepts explored in this review, and their relationship to the disease triangle model that depicts the interplay between host, pathogen, and environment (social, natural, physicochemical). Numbers indicate relevant sections.

Despite progress made since the discovery of the microbiological origins of Lyme disease, fundamental questions and controversies remain. On the subject of pathogenesis, the objective of this review is to consolidate and reconcile some of the key concepts in *Borrelia* and host biology that frame our understanding of the disease, while identifying opportunities for development. Major themes are outlined in Figure 1. Topics that are relevant to this dialogue but beyond the scope of this communication include the ecological and entomological drivers of disease, wildlife biology, prevention and prophylaxis, routes of transmission, co-infections, detailed physiology of LD and its sequelae, clinical findings, and case management. Indeed, this review is not intended to provide diagnostic guidelines or treatment recommendations, but rather seeks to explore the interface between microbiology and human disease.

## 2. Detecting and Characterizing Lyme Disease

From the perspective of both clinical care and biomedical research, it is imperative to be able to identify disease cases accurately, and also to monitor the trajectory and response to intervention. The diagnostic workup for LD traditionally involves an assessment of the patient’s risk, objective signs (such as an EM) and symptoms, supported by laboratory findings where appropriate [27]. In practice, this can be confounded by a number of factors, some of which are described below. Clinical microbiology is additionally hindered by the relatively low spirochetemic burden associated with the disease [28], and the fastidious nature of the pathogen [29], both of which pose problems for the recovery and propagation of live *Borrelia* from clinical specimens. Thus, our understanding of the microbial determinants of disease has been restricted in part by limitations imposed by culturing this microorganism. 

### 2.1. Diagnostic Challenges

The ideal diagnostic test for LD should be sensitive (to avoid false negatives), specific (to avoid false positives) and indicative of disease stage. In particular, it should delineate active infection, past exposure and re-infection. These objectives are not being met by the conventional approach.

However, in a review of 16 guiding documents for the diagnosis of LD originating from 7 countries, recommendations were found to consistently endorse two-tiered serology as the conventional laboratory test for all stages of LD except for the early dermatological manifestation of EM, where a clinical diagnosis is considered appropriate [30]. Two-tiered serology is an immunological technique that detects host anti-borrelial antibodies (IgG and/or IgM), traditionally beginning with an enzyme immunoassay (EIA) or equivalent, followed by an immunoblot if the first step is positive or equivocal [27]. Following a positive EIA, *Borrelia* proteins are electrophoresed and probed with host immunoglobulins, and the resulting banding pattern is evaluated against published criteria endorsed by relevant governing bodies to ultimately arrive at a binary outcome (positive or negative). Although the two-tiered technique is implemented internationally, the specific conditions, such as the antigens used for testing and the interpretation criteria, may differ by geographic region to best represent the relevant *Borrelia* species and prevalent immunological response [31]. The American Centers for Disease Control and Prevention (CDC) interpretation criteria exclude OspA (31 kDa) and OspB (34 kDa) as interpretable bands in the immunoblot step due to reported low levels of detection in the diseased population (except in cases of longstanding Lyme arthritis and late neurological disease), and to ensure that those individuals previously vaccinated for Lyme disease remain seronegative [32]. However, the elimination of OspA and OspB from the test despite their reported resurgence later in disease could bias against the potential for positive serology results in patients with late stage LD. In a meta-analysis of North American research, conventional two-tiered serology was reported to be 46.3% sensitive in early localized, 89.7% in early disseminated and 99.4% in late LD. However, these categories are based on clinical presentation only, and may not encompass the entirety of LD cases, thereby biasing results to represent only those subjects with objective manifestations [33]. Comparatively, a meta-analysis of serological testing in European patient cohorts showed sensitivity of 50% for those presenting with EM rash, 77% for neuroborreliosis, 97% for acrodermatitis chronica atrophicans and 74% for unspecified Lyme borreliosis [31]. Various iterations of the two-tiered approach are under active investigation to optimize sensitivity and specificity [31,33]. Interestingly, the CDC recently expressed support for a modified two-tiered serological test that implements a second EIA rather than the immunoblot, as an acceptable alternative to traditional two-tiered serology [34]. 

Although optimization of serology is beneficial, there are several inherent limitations of immunological techniques that cannot be overcome. Notably, serological testing requires a host immune response, which can take weeks to develop, and serology is an indirect indication of exposure to the bacteria, rather than a direct readout of active infection. There are additional microbiological and host considerations such as immune modulation and evasion [35] (discussed in Section 4.2), *Borrelia* biodiversity (Section 4.1), antigenic variation [36,37], and recurrent IgM and IgG responses [38,39] that add to the complexity of serological testing in LD. Extrinsic factors including early antibiotic exposure have likewise been found to influence the humoral response [40]. One study that compared pre- and post-treatment serology in patients presenting with EM found that 39.4% of participants were seronegative at both timepoints [41]. Moreover, the magnitude and diversity of the B cell response to *B. burgdorferi* has been correlated to a faster resolution of clinical symptoms [42]; therefore, research that relies on adaptive immunity to classify patients according to serostatus could bias the sample toward less severe manifestations than those experienced by the broader Lyme population. For patients who do seroconvert, the conventional diagnostic paradigm is unable to discern past exposure from active infection, which is problematic for the clinician or researcher attempting to determine residual infection. 

Due to the complexity and limitations of serological testing in LD, other diagnostic modalities are under active investigation [33]. These include techniques that measure host T cell response [43], and borrelial antigens shed in patient urine [44]. The common goal of many of these emerging approaches is to directly detect the presence or absence of *Borrelia* in a sensitive and specific manner. These evolving techniques have begun to uncover a substantial number of cases where there is evidence of active or previous *Borrelia* infection, alongside negative serology [44,45,46,47,48,49,50]. However, the majority of these approaches are not yet widely adopted in the clinic, but have been useful in research to learn more about the mechanisms of disease at various stages. 

### 2.2. Classifying Lyme Disease

The terminology that describes the progression of LD and its varying manifestations is central to framing and interpreting research questions, yet it has also been a topic of confusion and controversy. Thus, we will review common disease definitions for the purposes of clarity. The goal is to provide relevant information to gain a broader understanding of how laboratory findings and clinical presentation shape conventional LD classifications, and how research is ultimately impacted.

Typically for clinical and research purposes, LD is described in three stages: early localized, early disseminated, and late LD, although other designations such as chronic and post-treatment have also gained traction. The latter categories encompass protracted cases of LD in which patients experience ongoing symptoms and do not fall within one of the strictly defined early or late categories. 

**Figure 2:**
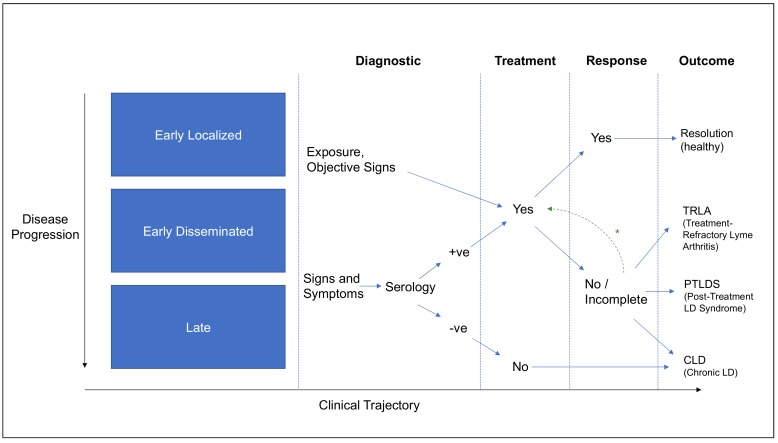
Schematic representation of the stages of Lyme disease, associated clinical decision points commonly applied, and possible outcomes. * Under specific conditions outlined in the IDSA guidelines, individuals with ongoing signs of Lyme disease may be re-treated.

Spirochetemia is detectable in 45% of patients with early LD, indicating that roughly half of early localized *Borrelia* infections undergo demonstrable hematological dissemination [51]. With disseminated infections, patients can experience signs and symptoms that depend upon a suite of factors such as geography, infecting species, host predisposition, and treatment history, as discussed in subsequent sections of this review. In North America, around 60% of untreated patients develop joint swelling and pain, 15% develop neurologic symptoms, and a smaller proportion develop cardiac complications [52,53]. Even when treated in an ideal manner, it is estimated that 10%–15% of patients develop post-treatment symptoms [54]. In Europe, early neuroborreliosis (10%–20% of symptomatic patients), Lyme arthritis, lymphocytoma, multiple erythemata (less frequent) and carditis (less frequent) are observed [55]. Long-term presentations can include acrodermatitis chronica atrophicans, lymphocytoma, chronic arthritis (rare in Europe), encephalomyelitis and chronic neuroborreliosis (rare in Europe) [55]. Similarly, in China, it has been estimated that 10% of cases may develop into chronic infections over 2 to 17 years without treatment [56]. A comparison of European and North American disease manifestations can be found in [52], and the associated microbiological determinants are described in Section 4.1. Current diagnostic tests are unable to differentiate definitively between the various stages and presentations of disease; thus physicians, researchers and patients rely on clinical definitions instead to delineate LD. When considering the forthcoming definitions, there is an important distinction to be made between surveillance definitions, clinical diagnoses, and microbiological/pathogenic understanding. These distinctions are captured in Table 1 and Table 2, and in Figure 2, to describe the current state of knowledge around the terms used to classify LD [55]. 

The stages and presentations of LD have been defined by organizations worldwide and these descriptions have previously been graded for methodological quality [30,57]. The European Centre for Disease Control and Prevention (ECDC) provides a case definition for Lyme neuroborreliosis, which relies on a combination of pleocytosis in cerebrospinal fluid (CSF), intrathecal Lyme antibodies, and isolation of Lyme *Borrelia* or nucleic acid detection in CSF. Other manifestations of Lyme Disease are not described by the ECDC. In North America, the 2017 CDC case definitions for early and late LD (outlined in Table 1) are commonly referenced in a wide range of contexts, including biomedical research and clinical practice, despite CDC recommendations that their case definitions are intended solely for surveillance purposes [58]. In each of these surveillance definitions, an EM and/or positive laboratory evidence is required, yet two-tiered serology is only 46.3% sensitive in early LD. Additionally, EM presents in only 60–80% of cases, and of those, it is estimated that only 72% are accurately identified by general practitioners, further hindered by the fact that only 9% of EM present as a classic bullseye with central clearing (CDC, 1990) [33,59,60,61]. Concerns have also been raised about EM appearance and recognition—or lack thereof—in different complexions, and the implications for underdiagnosis [62], particularly as reference images predominantly depict Caucasians. Therefore, it has been estimated that only 10% of total LD cases are reported using these strict surveillance definitions, emphasizing the need for physicians to use clinical judgement, and for researchers to expand the scope of studies [5,63].

#### Protracted Lyme Disease: Defining Chronic and Post-Treatment Conditions

Beyond early and late LD, protracted disease definitions become nebulous and inconsistent, further confounded by the changing use of language over time. The category of post-treatment Lyme disease syndrome (PTLDS) has evolved out of a need for a standard definition that can be used to categorize and research LD patients who have been treated and remain symptomatic. There are specific conditions required to meet the definition of PTLDS, since this category only encompasses patients treated with a recommended regime, who experience a resolution of objective symptoms and onset of a set number of subjective symptoms within a specific time frame. The Infectious Diseases Society of America (IDSA) guidelines describe subjective symptoms of PTLDS as fatigue, widespread musculoskeletal pain and cognitive difficulties, resulting in reduced levels of occupational, educational, personal or social activity [66]. Comparatively, to meet the requirements of the operationalized definition of PTLDS presented by Aucott and colleagues, patients must experience one of the following: higher level of fatigue than pre-infection, three or more areas of musculoskeletal pain, or difficulty finding words/concentrating or memory, in addition to a composite T-score of <45 on the 36-Item Short Form Health Survey (SF-36) [54], which evaluates health concepts across eight domains [71]. Additionally, the definitions identify exclusion criteria that eliminate individuals from the PTLDS classification based on active coinfections, co-morbidities, or pre-existing underlying conditions associated with fatigue or pain. Although the original IDSA definition also proposed excluding patients with positive culture or PCR result post-treatment [66], these criteria have not been explicitly adopted by Aucott and colleagues [54].

The term ‘chronic Lyme disease’ (CLD) has been a subject of debate throughout the scientific literature, medical practice, and social landscape of LD. CLD has been critically described by one source as the experience of persistent pain, fatigue and neurocognitive impairment in patients who do not have previous evidence of acute Lyme disease [70]. This is a challenging paradox since acute Lyme disease diagnosis is imperfect within itself. Indeed, CLD has also been used interchangeably with objective late LD and post-treatment LD, further complicating interpretations and defying standardization.

Currently, CLD is most often used as an umbrella term to describe individuals suffering from protracted illness suspected or proven to be LD, who do not fall into another category. Various labels have been proposed in the literature to help parse the complexities of CLD cases. These descriptors also demonstrate the extensive variability seen within the CLD classification. Patrick et al. proposed that ‘alternatively diagnosed chronic Lyme disease syndrome’ (ADCLS) encompasses CLD patients who have been diagnosed on clinical grounds and who also have a positive test result from a non-reference laboratory. These authors also describe ‘seronegative Lyme disease’ for patients who are diagnosed based purely on clinical grounds, emphasizing that seronegative LD is controversial outside of early LD [72]. Expanding upon these designations, Stricker and Fesler further suggested that CLD encompasses both treated (CLD-T) and untreated (CLD-U) patients. By its original IDSA definition, PTLDS implied clearance of the infection followed by a post-septic syndrome; comparatively, CLD-T includes patients who were treated for LD with an antibiotic regime that was inadequate to clear all *Borrelia,* leading to a persistent infection [73]. Presently, CLD-T and PTLDS cannot be differentiated in patients because there are no reliable biological correlates to confirm the presence or absence of active ongoing infection. 

Despite the confusion over terminology for protracted manifestations of LD, tools are being developed to parse through these complexities. The Horowitz Multiple Systemic Infectious Disease Syndrome (MSIDS) Questionnaire consists of four sections: symptom severity, Lyme Incidence scale (history of exposure), ranking of physical and mental health status, and the Common Lyme symptoms score. Each question is weighted and incorporated into a final score that ostensibly classifies patients into one of three categories: unlikely, possible, or probable Lyme disease. Total MSIDS scores have been shown to differ significantly between confirmed LD (EM and/or positive test results) and self-identified healthy individuals [74]. Although the potential of this instrument to discriminate between LD and other diseases has not been demonstrated, the questionnaire is intended to provide a holistic view of patient history and symptom set, and could therefore have utility both in research and in the clinic. Recently, another instrument was developed to assess and track the burden of multi-system symptoms associated with LD. Although it does not purport to be a primary diagnostic aid, and cannot distinguish between PTLDS and traumatic brain injury or depression, the General Symptom Questionnaire–30 (GSQ-30) is positioned as a monitoring tool for clinical and research use [75]. The GSQ-30 evaluates four domains relevant to LD (pain/fatigue, neuropsychiatric, neurologic, and viral-like symptoms), and is sensitive to changes in the patient’s wellbeing over the course of treatment. 

### 2.3. Implications for Research

For the purposes of standardization, consistency, and data integrity, the strict surveillance case definitions (Table 1 and Table 2) are usually applied in human LD studies to minimize the inclusion of false-positive subjects. Thus, much of what is known about borrelial pathogenesis is predicated on classical presentations of the disease. Although it has been argued that a conservative case definition is a necessary quality control measure to generate valid findings that may be applicable to a broader population, including less typical manifestations, the biological foundation of this assumption has not been tested. As discussed above, the magnitude of the adaptive immune response not only predicts disease duration [42], but may also be modulated by factors such as antimicrobial intervention [40,41]. Thus, even within the relatively well-defined PTLDS umbrella, which only includes definitively-diagnosed LD, seropositive and seronegative cohorts exist [41], and may be biologically distinct in relevant ways. This diversity calls into question the validity of statistical inferences made from textbook presentations to a more heterogeneous population. Indeed, the role of adaptive immunity both in clearing infection and determining diagnostic serostatus may confound interpretations, which provides additional impetus to identify more robust and informative ways of defining cohorts of interest.

It is also imperative to note that for both PTLDS and CLD, the microbiological correlates of the disease are intensely debated. Hypotheses to account for longstanding illness include immune dysfunction (including autoimmunity, discussed in Section 3.1), sustained inflammation and/or reactivity to pathogenic debris, co-infections, and ongoing colonization with persistent *Borrelia* (Section 5.1). Critiquing the evidence of protracted disease mechanisms is a topic of future correspondence, although we review some of the foundational concepts in the following sections.

Due to the current lack of biological indicators to distinguish between acute, treated, and ongoing infection, it is challenging to define and delineate these patient groups, which has heightened the controversy and confusion surrounding ‘chronic Lyme disease’. It is important to consider the challenges and variable usage of these disease stage definitions when evaluating the scientific literature, and critically assess the conclusions accordingly. Effective and consistent communication is integral to progress in the field, and developing common terminology reflective of biological understanding would greatly benefit LD stakeholders across all sectors.

## 3. Pathogenicity: Host Predisposition and Defense 

Considering the variable disease presentations and outcomes discussed above, an intriguing question is why some LD patients exhibit the classical pattern of disease progression with the appearance of EM and good response to antibiotic intervention leading to a full recovery, whereas others do not respond to the treatment and experience life-long complications. Moreover, are there any factors that would predispose some patients to develop the neurological symptoms, known as neuroborreliosis, whereas other patients develop skin disorders, Lyme carditis, or arthritis? These questions are particularly important since the answers would allow clinicians to make more accurate prognoses and adjust the treatment regimens to be personalized to each individual patient. In this section, we consider several host factors that could play a role in making some patients more susceptible to LD than others and predispose them to different disease progression.

### 3.1. Genetic Susceptibility to Disease and Autoimmunity 

In general, when a disease can be studied in an animal model, there are multiple benefits such as the use of different molecular biological and genetic tools. Fortunately, Lyme borreliosis can be induced in laboratory mice, rats, and rhesus macaques by intradermal, intraperitoneal, or intrathecal injection of *Borrelia burgdorferi* isolates [76,77,78], or by infected tick feeding [79]. Rhesus monkeys probably represent the animal model closest to the human disease since the manifestations of LD in this model include the EM, neuroborreliosis, mononeuritis multiplex, and arthritis. However, this model is not suitable for quick genetic manipulations and is expensive. Therefore, the most widely used animal model for LD is the mouse [15]. It has been observed that the natural course of infection is not the same in different genetic backgrounds of mice, and that some strains are more resistant to the disease than others. For instance, BALB mice develop only mild arthritis, whereas C3H mice develop spirochetemia followed by severe polysynovitis and carditis 2–4 weeks after the intradermal inoculation [80]. Moreover, infant BALB mice or C.B.-17 mice with severe combined immunodeficiency (BALB-congenic) are similar to C3H mice and develop severe pathology [77,81,82], suggesting that susceptibility to disease has an immunological component. Also, around 60% of human patients in North America [52] and up to 24.5% in Europe [83] develop migratory joint pain that results in chronic polysynovitis, characterized by synovial lesions similar to other types of chronic inflammatory arthritis such as rheumatoid arthritis [53]. Yet, only a relatively small percentage of LD patients develop chronic objective arthritis, which suggests that there could be host-related factors that determine susceptibility to the natural course of disease. This idea is further supported by the proposed animal model of chronic Lyme arthritis in the CD28 knock-out (CD28^−/−^) mice, which also suggests the involvement of host immunity in generating different responses to infection [84]. In this model, an active immune response to the pathogenic antigens remains intact; however, the immunoregulatory pathway involving CD4^+^CD25^+^ regulatory T cells (Treg) is compromised. When this model is infected with Bb, the acute inflammatory stage of the Lyme arthritis is indistinguishable from the control mice (CD28^+/+^), but the incidence rate of the chronic arthritic manifestation is much higher.

There are similarities between some musculoskeletal presentations of Lyme disease and rheumatoid arthritis. The latter has an autoimmune component and is associated with the human leukocyte antigen (HLA) system encoding major histocompatibility complex (MHC) Class II proteins, specifically the HLA-DR isotype (as reviewed in [85]). Thus, studies have been conducted to probe the association between susceptibility of LD patients to chronic arthritis and certain HLA-DR serotypes. In the pioneering work by Steere and co-workers on 10 patients with chronic Lyme arthritis, it was found that, in contrast to rheumatoid arthritis (higher prevalence of HLA-DR4 serotype), 7 patients had the HLA-DR2 and 4 had the HLA-DR4 serotype [53]. In their next study, they conducted a more detailed investigation on a larger group of 130 participants with various manifestations of LD [86]. In this larger cohort, they found that HLA-DR4 was more prevalent in the patients with chronic arthritis: 57%, compared to 23% in patients with arthritis of moderate duration, and only 9% in those with short duration. Additionally, they found a secondary association with HLA-DR2, namely, in the patients not containing HLA-DR4, HLA-DR2 was found in 75%, 50%, and 20% of chronic, moderate and short duration arthritis cases, respectively. They concluded that overall in 89% of patients with the chronic pattern of arthritis, either HLA-DR4 or -DR2, or both, were found and appeared to act as independent dominant susceptibility markers. Intriguingly, HLA-DR4 was the only serotype significantly associated with treatment-refractory Lyme arthritis (TRLA) [86], with an increased frequency of the HLA-DRB1*0401 allele in the non-responsive cohort [87]. These patients were also found to develop strong IgG responses to *Borrelia* outer surface proteins, OspA and OspB, near the onset of their arthritic attack [88]. Based on these findings, the authors proposed that, in genetically-predisposed individuals, an arthritogenic antigen of *B. burgdorferi* possesses molecular mimicry to a host component causing an autoimmune response that continues even after the microorganism has been killed, thus making the arthritis unresponsive to the antibiotic treatment. When this hypothesis was tested, a bacterial immunodominant epitope associated with HLA-DRB1*0401 was identified as an OspA peptide (aa 165-173) [87] with sequence homology to a region of the human leukocyte function-associated antigen-1 (hLFA-1) (aa 332–340). This region of hLFA-1 was demonstrated to act as a partial agonist to OspA-specific T cells, and resulted in a similar immune response and in ability of HLA-DRB1*0401 to present this autoantigen even after antibiotic intervention, thus supporting further the idea of an autoimmunity component in the treatment-refractory Lyme arthritis [89]. 

More recently, another group has proposed that a Bb infection could trigger an autoimmune thyroid disease (AITD) in patients with certain HLA-DR alleles [90], and as reviewed in [91]. Their results demonstrate homologies between the four thyroid autoantigens and Bb proteins, one of them being OspA [90], hence providing more support for the role of autoimmunity in the treatment-resistant Lyme arthritis. This idea has been also supported by the study in the CD28-/- mouse model (mentioned above) with the DR4^+/+^MHCII^−/−^ background [92]. In these animals, chronic arthritis did not resolve after antibiotic treatment, contrary to the control wild-type animals which reacted well to treatment.

Yet, it is important to mention that, although the autoimmunity in patients with antibiotic-resistant Lyme arthritis is supported by multiple studies, it remains unclear why the association between any specific HLA type and immune response to OspA or hLFA-1 in the patients who developed arthritis post recombinant OspA-Lyme vaccination was not observed [93]. It is also imperative to note that, by definition, the TRLA documented in these studies is distinct from post-treatment Lyme disease syndrome (PTLDS) described in Section 2, as the latter assumes resolution of objective manifestations, and focuses on a broader constellation of symptoms and functional impairment. TRLA is therefore identified as a distinct outcome in Figure 2. When the OspA-LFA cross-reactivity hypothesis was evaluated in a chronic LD patient cohort exhibiting a spectrum of symptoms of more than three months’ duration, no association was found between T cell response to LFA and clinical outcome [94], suggesting that the disease mechanism may be unique to TRLA.

### 3.2. Influence of Host Diet and Lifestyle: Hypercholesterolemia and Eicosanoids

Still focusing on the host response to infection, there are factors beyond genetic predisposition that can influence the severity of disease. It is well-known that different comorbid conditions represent an additional disease burden and could lead to a different course of disease, and/or interfere with the efficacy of medical intervention. One such factor could be blood cholesterol level. Cholesterol is involved in the synthesis of the sterol hormones, and is also an essential structural molecule responsible for eukaryotic cell membrane fluidity and formation of microdomains. There are few prokaryotes that require cholesterol for their membranes or metabolism; however, several bacteria, including *Borrelia burgdorferi*, contain cholesterol derivatives, such as cholesterol glycolipids [95,96]. Interestingly, although cholesterol glycolipids represent ~23% of total Bb lipids [96] and are required for growth, *Borrelia* cannot synthesize cholesterol and depends on the host to acquire it [97]. It is logical to suspect that in hypercholesterolemic patients, where cholesterol is more accessible, it would be easier for Bb to acquire it and therefore to cause more severe symptoms or faster dissemination with poorer prognosis. Indeed, it has been demonstrated in an animal model of Lyme disease that deficiency of one of the key components of the efficient cholesterol transport and metabolism, ApoE protein or LDL receptor, leads to higher spirochetal load in the blood and joints of the hypercholesteremic animal and more severe inflammation [98]. 

Recently, another study evaluated the effect of apheresis on the symptoms and signs related to Lyme disease [99]. This study analyzed the profile of lipids in the blood of patients with proven history of Lyme disease before and after apheresis, and probed the association between the level of cholesterol and symptoms of the disease. The study demonstrated that a reduction in the concentration of blood inflammatory lipids correlated with improved symptoms and reduction in the levels of acute-phase inflammatory marker, C-reactive protein (CRP). However, due to some technical constraints of this study, the authors could not conclude that there is a causal effect of the elevated levels of blood cholesterol and/or other lipids on severity of LD. A better-designed, randomized controlled study is required to answer these questions, and to allow further investigations into potential medical intervention.

We also cannot dismiss the potential role of other lipids in the development and progression of Lyme disease. In fact, eicosanoids, the metabolic derivatives of arachidonic acid (AA) and/or eicosapentaenoic acid (EPA), are involved in the induction and resolution of the inflammatory response. There are three main pathways in the enzymatic metabolism of AA and EPA, which involve cyclooxygenases (COX-1 and COX-2), lipoxygenase (LOX), and cytochrome P450 (CYTP) to produce prostaglandins (PGs), thromboxanes, leukotrienes, lipoxins and epoxyeicosatrienoic acids [100]. Indeed, EPA and AA, which are omega3- and omega6-polyunsaturated fatty acids (PUFA), respectively, compete for these enzymes in the same metabolic pathways. It was shown that the expression of COX-2 in the joints of Bb-infected mice increased two weeks post-infection, and remained elevated for two months [101]. Interestingly, treatment with COX-2-specific inhibitor as a mimic of non-steroidal anti-inflammatory drugs (NSAIDs) alleviated the arthritic symptoms without interfering with the immune response [*ibid.*]. However, a later study that utilized the COX-2^−/−^ knockout mouse model did not confirm the direct involvement of COX-2 levels in the development of Lyme arthritis, and, in fact, there was a significant delay in the resolution of symptoms in this knockout model [102]. Yet, the overall results supported the finding of the previous study that arthritic inflammation is uncoupled from the immune response and Bb clearance from the tissue. The role of eicosanoids in the development and resolution of Lyme arthritis was studied by comparing the eicosanoid lipids profile between joint tissues from arthritis-resistant and arthritis-susceptible mice during the course of Lyme arthritis [103]. The authors found that a prostaglandin, PGD2, which is a COX pathway metabolite, did not increase in the arthritis-resistant mouse strain, but was significantly elevated in the susceptible mice. Since PGD2 is one of the proinflammatory prostaglandins, this difference is a potential clue in identifying the source of susceptibility to arthritis, and requires further research into the potential role of NSAIDs in combating inflammation in LD. 

The eicosanoid profile seems to be important for the development and resolution of Lyme arthritis symptoms, so the effect of a diet rich in omega6- or omega3-lipids has been studied. There is a general consensus that omega3-PUFA compete with omega6-PUFA in the metabolic pathways involving the same enzymes, namely, COXs, LOX and CYTP, and their metabolites (omega3 eicosanoids) are less active and are more anti-inflammatory than their omega6 counterparts. In fact, a diet rich in omega3-PUFA is associated with health benefits in prevention and outcome of different diseases [104,105]. Dumlao et al. have evaluated the effect of diet rich in omega3- and omega6-PUFA on Lyme arthritis in a mouse model [106]. As expected, the authors observed a shift in the profile of eicosanoid metabolites with an increase in the anti-inflammatory markers in an omega3-PUFA rich diet, whereas pro-inflammatory metabolites were increased in an omega6-PUFA rich diet. Surprisingly, they could not detect any significant differences in the severity of Lyme arthritis in mice fed with these two diets, therefore indicating that the eicosanoid profile is not the only factor influencing the severity of symptoms. Altogether, it remains unclear how, if at all, the metabolites of omega3- and omega6-PUFA are involved in the inflammatory and immune response in LD. Further studies are required to better understand the role of eicosanoids in disease progression and in the ability of NSAIDs to fight the symptoms or even aid in the treatment and resolution of disease. 

## 4. Pathogenicity: *Borrelia* Virulence

The concept of virulence, or infection causing disease, has classically been centered on the microorganism and its host-defeating properties. Yet increasingly, it is being recognized that virulence cannot be modelled in a simple reductionist way, because it is relative to, and indivisible from, the host–microbe association and the context in which it occurs [107]. The definition of virulence as “a complex, dynamic, and changeable phenomenon that includes both host and microbial factors” [107] is particularly relevant to Lyme *Borrelia*. As a tick-vectored zoonotic agent, *Borrelia* encounters a variety of biotic environments that require unique adaptations for survival. As suggested in Section 3, it has become apparent that the interplay between vector, spirochete, and mammalian host determines the severity and manifestation of Lyme disease, in ways that are only beginning to be delineated. For example, cultures of laboratory-propagated *Borrelia* that have lost pathogenic properties in mice have been found to reacquire them when the strain is passaged through the *I. scapularis* vector [108]. Similarly, the extent of tick feeding influences the infectious potential of the *Borrelia* that it harbours, such that the spirochetes in a starved tick are markedly less capable of colonizing a host [109]. This phenomenon is independent of the underlying mechanical requirement for *Borrelia* to translocate from the tick midgut to the salivary glands to facilitate transmission, and instead appears to relate to bloodmeal-based priming of yet unknown virulence factors [109].

Although members of the Lyme complex are well-documented human pathogens, seroprevalence surveys that suggest exposure to the spirochete in the absence of remarkable disease allude to the possibility of asymptomatic infection [110]. Meanwhile, reported recovery of Lyme *Borrelia* from chronically ill, antibiotic-treated, seronegative patients [111] challenges some of the conventional discourse around the disease.

### 4.1. Borrelia Biodiversity and Disease

The number of genospecies belonging to the Lyme borreliosis complex continues to expand, while the human pathogenicity of many of them remains unknown [112]. At least 21 confirmed or presumptive species have been identified [112], including those routinely found in patients (*B. burgdorferi* s.s. (N.A., Europe) *B. garinii*, *B. bavariensis*, *B. afzelii* (Europe, Asia) and *B. spielmanii* (Europe), and others that have been discovered more recently and/or have limited documentation in the clinical setting (*B. lusitaniae*, *B. valaisiana*, *B. japonica*, *B. kurtenbachii* [113], *B. bissettii* [114], and *B. mayonii* [115]) [9]. The capacity of conventional serological testing to detect the more obscure genospecies is largely unknown. However, case and cohort study reports of variable serological findings associated with divergent species warrant additional investigation into the performance of existing tools, and consideration when developing novel diagnostic platforms. The recent report of live *B. bissettii* recovered from a chronic seronegative patient in the United States [111,116] emphasizes the need to revisit laboratory diagnostic capabilities as the breadth of the Lyme complex increases.

#### 4.1.1. Genospecies, Geography, and Disease Manifestation

A decade after the bacterial origins of LD were first traced to a novel spirochete, *Borrelia burgdorferi*, evidence of similar yet distinct isolates from North America and Europe prompted a further division into the three genospecies that are most synonymous with LD—*B. burgdorferi* s.s., *B. garinii*, and *B. afzelii* [117]. In Europe, where all three major pathogenic species are endemic, resolution of lineages also began to reveal associations between symptom presentations and the genotype of recovered organisms, suggesting that pathogenicity and tissue tropisms differed between species [118]. The findings implicated *B. afzelii* as the dominant driver of the chronic skin presentation acrodermatitis chronica atrophicans, which, like the genospecies, is rare in North America [119]. Conversely, *B. garinii* was most frequently found in neuroborreliosis, and *B. burgdorferi* came to be associated with arthritic manifestations [120]. More recent analyses have also implicated *B. bavariensis* in neuro-Lyme [121,122]. Even the EM rash, which is often considered a hallmark feature of local infection in the human host, appears to be disproportionately affiliated with *B. afzelii* in European samples [123].

Intriguingly, LD in North America is characterized by many of the same clinical features as its European counterpart, including dermatological, arthritic, cardiac, and neurological manifestations, although it has long been assumed that *B. burgdorferi* s.s. is the sole etiologic agent on the continent [124]. This assumption has been challenged recently by the discovery of *B. mayonii* [115] and recovery of other rare genospecies from American clinical isolates. Nevertheless, many studies have attributed diverse American symptom sets to a single genospecies. 

Clinical and mechanistic studies broadly support the observation that North American LD can indeed be physiologically distinct. When EM and disease trajectories were compared between patients in Austria and the United States between 2001 and 2004, striking differences were noted. Austrian skin biopsies positive for *B. afzelii* were associated with a slowly expanding rash and few, if any, additional symptoms [125]. In contrast, *B. burgdorferi*-driven lesions in American patients enlarged rapidly, contained higher levels of chemokine and cytokine mRNA, and presented with a median of four other signs and symptoms [125]. Surprisingly, these associations held up when comparing the same genospecies, *B. burgdorferi* s.s., isolated from clinical cases in North America and Europe [126]. *B. burgdorferi* from the American Eastern seaboard was found to have higher inflammatory potential and drive more severe early disease than Slovenian isolates of the same species, which instead resembled infection patterns characteristic of *B. afzelii* or B. *garinii* [126]. Long-term prognosis of the cohorts was not documented. 

Despite these findings, it cannot be concluded that North American Lyme *Borrelia* are homogenous. Indeed, it has been noted that the clinical presentation of Lyme can vary considerably between patients in a single American region [127]. Clearly, although genospecies designations roughly capture symptom sets, they fail to accurately represent the full complexity and nuance of the clinical picture. 

#### 4.1.2. Serotypes and Invasion

Higher-resolution methods of categorizing Lyme *Borrelia* have proven useful in deciphering phylogenetic relationships of strains, and probing their association with disease. A number of classification schemes are currently in use, including MLST, RST, and OspC profiling, each with their own implications. Multilocus sequence typing (MLST) of *Borrelia* typically evaluates eight predetermined housekeeping loci on the linear chromosome, and compares sequences against those in a database to assign an eight-integer allele profile [128]. Ribosomal spacer typing (RST) resolves *B. burgdorferi* s.s. into three types (RST1, 2, or 3) based on the fingerprint produced by amplification and restriction endonuclease digestion of an rRNA intergenic spacer region [128]. Finally, OspC-based genotyping involves sequencing the gene for Outer Surface Protein C and assigning it to a major group or type depending on the degree of identity with other alleles [128]. The OspC groups are defined as containing allele sequences that differ by less than ~2%, while differences between groups exceed 8% [129], and may be as high as 35% [130]. 

OspC is a strategic candidate on which to base a typing system. The 22 kDa lipoprotein, localized to the bacterial outer membrane, is induced upon tick feeding and expressed during early mammalian infection [131], at which point it is a key virulence factor enabling vector-to-host transmission and colonization [132]. Variable participation in longstanding murine borreliosis has also been proposed based on OspC expression profiles following experimental infection [133]. Although its function is incompletely characterized, it appears to have roles in immune modulation through activities such as macrophage evasion [134] and complement pathway inhibition [135]. *OspC* is a highly polymorphic locus encoded on the cp26 plasmid, which is a ubiquitous feature of the genome [136]. Indeed, estimates suggest that OspC is “at least an order of magnitude more variable” than other genes of the Lyme complex [137]. The potent immunogenicity of epitopes in the variable regions of the protein [138] suggests that the major OspC groups represent distinct borrelial serotypes [127,139].

OspC typing efforts have revealed a number of such groups, also referred to in the literature as types/serotypes, lineages, or clones, in recognition of the low degree of recombination [140]. For *B. burgdorferi* s.s. alone, there are upwards of 22 documented groups, of which at least 16 have been found in the northeastern United States [137,141]. Diversity can be high in endemic areas, as evidenced by the discovery of 11 OspC groups in a single sampling site in New York state [129]. Extending the analysis to *B. afzilii* and *B. garinii* in Europe raises the number of distinct OspC types to at least 69 [142]. Just as the groups are not evenly distributed geographically, they are not found in equal frequency among reservoirs, vectors, and human hosts; nor do they appear to contribute equally to disease [137,143].

From a medical standpoint, this disequilibrium delineates the virulence of OspC types based on rates of recovery from the environment and clinical specimens. Strains isolated from blood, cerebrospinal fluid, or tissues distal to the site of inoculation are considered invasive or capable of producing disseminated, systemic disease, whereas those only found at the inaugural EM lesion are restricted to local infection. Groups identified in vectors or reservoirs, but not detected in patients, are considered non-pathogenic to humans [140]. 

Findings from several investigations have identified at least 12 *B. burgdorferi* OspC types associated with disseminated human infection in the United States (A, B (RST1); F, H, K, N (RST2); C, D, E, G, I, M (RST3)) [140,141,144]. Among these, types A, B, K and I were recovered from more than 80% of the culture-positive invasive disease cases studied in one investigation, suggesting that they may be particularly virulent [141]. One consideration when interpreting these findings is their reliance on clinical culture, which is notoriously challenging and unreliable, particularly in disseminated disease. The potential for recovery bias to influence biodiversity assessments should not be overlooked, as viable non-cultivable organisms may be present but not accounted for. Disparity between culture and direct typing has previously been noted [145].

To this end, the application of new high-sensitivity techniques and deeper sequencing may further illuminate—or complicate—the mystery of *Borrelia* pathogenic potential. Recent work coupling PCR and mass spectrometry to detect and classify *Borrelia* directly from the blood of early LD patients without cultivation revealed novel genotypes, and in one case, a co-infecting borrelial genotype that varied over the course of disease in the presence of therapeutic antibiotics [146,147].

Beyond invasive capacity, additional clinical implications of OspC types have been investigated. One evaluation of diagnostic test performance using acute phase serum from patients with EM suggested that OspC grouping of the invading microbe may affect the sensitivity of the two-tiered approach [148]. Another American study focusing on Lyme arthritis discovered an overrepresentation of RST1 (OspC types A or B) in treatment-refractory arthralgia (TRLA), whereas other types were detected in antibiotic-responsive arthritis cases [149]. Serotyping may also help to explain earlier observations of distinct disease presentation on different continents. In the previously-referenced comparison of clinical *B. burgdorferi* s.s. isolates from Slovenia and the American Northeast, OspC analysis revealed a largely different collection of genotypes in the two locations [126]. Notably, of the four types implicated in invasive disease in North America [141], only one (OspC type B) was found in the Slovenian sample set [126]. Absent from that particular European sample was OspC type A, which has been associated with increased inflammation and more severe manifestations of Lyme disease [150].

Considering the pivotal role of OspC in establishing mammalian infection, it is reasonable to hypothesize that the OspC alleles themselves directly influence the physiological response and associated clinical trajectory of the disease. However, this hypothesis remains largely untested. The majority of mouse modelling and in vitro cytokine work has used representative strains of different groups instead of testing OspC variants in a uniform genetic background. Those studies that have compared recombinant OspC-type proteins have done so in search of physiologically-relevant binding partners that could begin to explain the difference in virulence. Using small subsets of OspC variants, investigators have found differences in affinity for plasminogen [151], a factor in extracellular matrix invasion, and the complement protein C4b, involved in both the classical and lectin immune pathways [135]. In the case of plasminogen, the strength of its interaction with four tested OspC proteins (types A, B, F, and H) corresponds to the invasive status of the respective *Borrelia* strains [151]. OspC also appears to play a role in bloodstream survival, and C4b binding of three OspC proteins (types A, B (*B. garinii* derived), and M) aligns with the reported serum-sensitivity of the associated strains [135]. Although these findings are intriguing, it remains unclear whether OspC alleles themselves are key drivers of clinical outcomes, or whether this locus is one component of a larger haplotype comprising other mechanistic determinants. 

Despite the underlying biochemical ambiguity, OspC typing has been useful in conjunction with other loci to delineate phylogenetic relationships between pathogen clusters and geography, thereby contributing to the broader understanding of LD epidemiology and the apparent explosion of the disease in recent decades. While *B. burgdorferi* s.s. OspC type A and B are both found in Europe and North America, where they are associated with disseminated disease, isolates of type B demonstrate continent-associated genetic polymorphisms suggestive of geographic subtypes, whereas type A is homogeneous at the loci investigated [152]. The lack of diversity in type A strains has been interpreted as evidence of recent, rapid, and wide dispersal of a high-virulence clone that appears to have originated in North America and moved trans-Atlantically [152]. These observations also suggest that regional presentations of Lyme disease characterized to date may be subject to change with the spread and selection of different serotypes. 

### 4.2. Host Colonization and Survival Strategies

As an obligate parasite that is incapable of synthesizing amino acids, nucleotides, and lipids de novo [14], *Borrelia* relies on its various hosts for survival. It has therefore evolved an arsenal of defenses to protect itself from metabolic stress and extrinsic attack (for example, by the innate and adaptive immune system), and it utilizes a repertoire of strategies to disseminate from the site of inoculation and invade distal tissues. In traditional reservoir hosts like the white-footed mouse, Bb infection most often manifests as a persistent, asymptomatic infection [15,153], whereas inbred laboratory models such as the C3H/He background suffer debilitating tissue pathologies [154], as discussed in Section 3.1. Longstanding infection has been described both in untreated model organisms and humans [155]. The interactions with the host immune system and other cells and tissues of the body are therefore thought to be key determinants driving the outcome. Although great strides have been made in elucidating mechanisms of transmission, dissemination, and persistence, there is much yet to be learned.

#### 4.2.1. Immune Modulation

**Figure 3:**
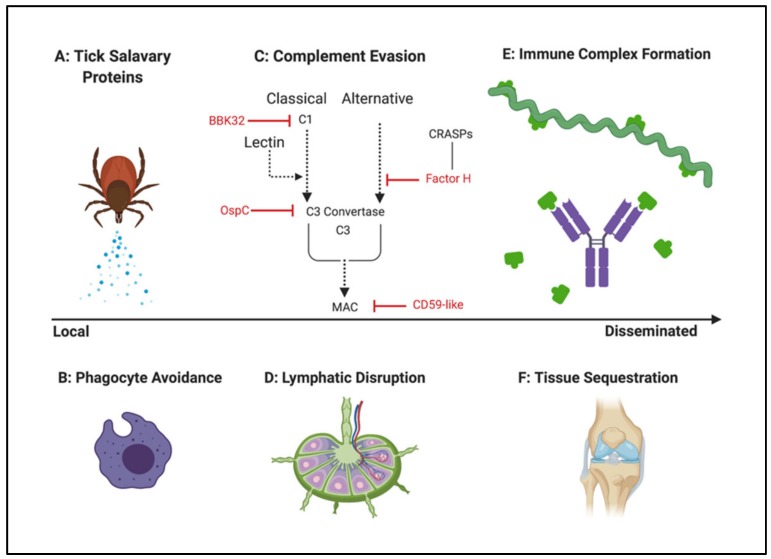
Immune evasion strategies used by *Borrelia* in the mammalian host, as discussed in the text.

Immune evasion strategies used by *Borrelia* to establish infection have been extensively reviewed elsewhere [35,156,157,158,159,160,161,162,163]. Thus, the detailed biochemistry of host subversion is beyond the scope of this communication. Nevertheless, no discussion of *Borrelia* pathogenesis would be complete without considering the many mechanisms that it employs to circumvent the natural defenses of its mammalian hosts. A visual summary of these tactics is presented in Figure 3. 

When *Borrelia* is initially transmitted from vector to mammal, some of the first factors that protect the spirochete from local attack are actually salivary proteins produced by the tick. They appear to suppress a number of host responses, including phagocytosis, cytokine release, complement activation, and immune cell recruitment and stimulation [159,164]. This capacity of tick salivary proteins to attenuate the host response has been found to protect *Borrelia* isolates that are otherwise serum-sensitive [165].

Protection conferred by tick proteins is not indefinite, however, and *Borrelia* requires autonomous strategies to survive and disseminate in a hostile new environment. Upregulation of bacterial virulence factors begins in the tick midgut upon feeding, when the spirochete is exposed to vertebrate blood [109]. There, OspC is among the proteins induced under the control of the RpoN-RpoS regulatory pathway [166], signifying a shift from tick colonization to vertebrate transmission. 

*Borrelia* strains that are serum-resistant possess inherent mechanisms to evade the mammalian complement system, a front-line defense that involves a cascade of host factors terminating in the assembly of a membrane attack complex (MAC) and bacteriolysis [167]. Several distinct strategies appear to account for borrelial avoidance of complement-mediated clearance, including recruitment of regulatory components, and direct interaction with complement effectors. In the first scenario, proteins on the surface of the microbe (for example, members of the CRASP, or complement regulator-acquiring surface protein, family) capture host inhibitors of complement (e.g., factor H) and use them to prevent deposition of complement proteins leading to borrelial destruction [167]. More recent findings also implicate borrelial proteins as direct antagonists of complement activation. In the case of lipoprotein BBK32, this is achieved by preventing catalysis of the C1 proenzyme of the classical pathway [168]. In contrast, OspC appears to oppose complement by disrupting the C3 convertase that is common to both the classical and lectin pathways [135]. Further downstream in the cascade, a borrelial protein ostensibly mimics host CD59, which prevents membrane attack complex (MAC) assembly [169]. These approaches, illustrated in Figure 3, appear to provide multiple points of resistance to complement-mediated clearance, conferring protection against the classical, lectin, and alternative pathways [161].

*Borrelia* has also been shown to modulate the activity of immune cells involved in both the innate and adaptive responses, in order to resist clearance. Murine models have revealed antiphagocytic mechanisms involving bacterial stimulation of macrophages to produce anti-inflammatory interleukin-10 (IL-10), which then appears to operate in an autocrine loop to suppress *Borrelia* uptake [170,171]. Subsequent work identified that OspC protects *Borrelia* from macrophage phagocytosis [134], although it is not yet known whether it functions as part of the IL-10 defense. *Borrelia* also has the capacity to resist the burst of reactive oxygen species produced by advancing phagocytes, via manganese superoxide dismutase antioxidation [163]. 

Lymphocytes and lymphatic infrastructure are likewise targets of *Borrelia* survival tactics, although the mechanisms of evasion have been somewhat elusive. Early accounts identified *Borrelia* and their extracts as potent lymphocyte mitogens, eliciting both specific and non-specific B cell proliferation and antibody production [172,173,174,175,176]. It was also recognized that the adaptive response was often insufficient to eliminate the pathogen from patients [172], and that polyclonal B cell activation and high levels of interleukin-6 (IL-6) observed in mice and in isolated human cells could be driving elements of the pathology [176]. Paradoxically, application of *Borrelia* cell extracts to cultured lymphocytes in the presence of other mitogens was found to yield a pronounced inhibitory effect on cell proliferation [177], which was also noted in animal models of live infection [178]. A similar response was observed by exposing cultured human lymphocytes to a canine Lyme disease vaccine composed of recombinant, non-adjuvanted, lipidated OspA. The inhibition elicited by OspA alone was more profound than that of the whole cell sonicate, suggesting that OspA may be a key component of the lymphocyte cell cycle block [179]. Another in vitro co-culture study reported Bb invasion and lysis of human B and T cells [180].

More recent work in mouse models has further probed the relationship between the lymphatic microenvironment of *Borrelia* processing, and the resulting adaptive immune response. As reviewed by Tracey and Baumgarth [35], a more complete picture has thus since emerged, portraying a vigorous but misguided host defense that confers some protection without fully eliminating spirochetes from murine tissues. The response appears to be mediated by borrelial invasion of the lymph node cortex, where the pathogen triggers lymphadenopathy and loss of functional lymph node architecture [181], essentially reducing T and B cell synchronicity [182,183]. Germinal centres (GC), which contribute to a mature and sustained immune response through the generation of robust antibody-secreting cells and memory cells, rely on T and B cell coordination [35]. Upon experimental infection with *Borrelia*, murine GCs were found to be delayed, abnormal, and short-lived, predicting the weak long-term humoral responses that have been documented in mice and human Lyme patients after antibiotic treatment [183,184]. Moreover, the immunosuppression observed in mice was not limited to the spirochetal infection, as an influenza vaccine co-administered with *Borrelia* also failed to induce a protective response [184]. Although the usual caveats apply about extrapolating murine data, this work provides a plausible mechanism of immune suppression that may be relevant to the human manifestations of the disease. 

Even though the adaptive defense against Bb is already suboptimal, it appears to be further subverted by the formation of immune complexes (ICs) that sequester antibodies [158]. Early observations of weak humoral response prompted speculation that sub-threshold immunoglobulin titer could result from the formation of antibody-antigen aggregates that are not recovered or accounted for during routine serological analysis [185]. Subsequent studies of ICs in human Lyme patients revealed several anti-*Borrelia* antibodies along with pathogen-derived proteins, of which OspA is the only antigen that has been definitively identified within the complexes [186,187]. Experiments in animal models support the hypothesis that OspA-specific antibodies can be generated early in the course of infection, but are enriched in complexes and may be undetectable without appropriate IC processing [188]. Indeed, recovery of *Borrelia*-specific ICs from human patients, who were otherwise seronegative by conventional assessment of free antibody, has been documented [185]. These findings, coupled with the observation that ICs diminish upon treatment, became the basis for a proposed modification to the serological diagnostic test that ostensibly increased sensitivity and improved the capacity to distinguish active infection from past exposure [189,190]. The concept was met with criticism, however [191], and did not appear to gain widespread traction. 

Overall, the microbiological implications of soluble antigens, ICs, and their involvement in *Borrelia* pathogenesis are intriguing, but not well understood. Outstanding questions remain around the origins and identities of the shed proteins that become antigenic cargo nucleating the complex, as well as their larger role in driving disease or defining its progression. Cell-free pathogen proteins are thought to arise from borrelial membrane vesicles that have been observed under various conditions [192], although immune-mediated disruption of the bacterial cell has also been postulated as a source of membrane proteins in host blood [193]. Likewise, the antigenic composition of ICs has not been thoroughly characterized. Sequestration of antibodies in ICs by decoy antigens is speculated to prevent effective opsonization and clearance of the pathogen [158], but has yet to be demonstrated experimentally. Thus, epitope shedding as a dedicated virulence mechanism is conceivable, and warrants further investigation. 

#### 4.2.2. Host Evasion

In addition to directly interfering with the function of the immune system, *Borrelia* employs parallel strategies to mount a stealthy invasion and avoid detection and destruction by the host. These cloaking mechanisms can shield the pathogen from host surveillance and defenses by reducing surface antigen presentation, changing the exposed proteome, or altering specific domains of select membrane proteins [158]. 

The unexpected finding that outer surface lipoproteins (OspA, B, and C) can be detected in the periplasm and appear to shuttle back and forth between the surface suggests that epitope exposure may be a regulated characteristic [131]. Antigenic variation involving cassette swapping at the *vls* locus is a well-documented phenomenon in mammalian infection that has also been reviewed elsewhere [194]. These strategies likely help Bb disseminate “under the radar” as they move through the vasculature and lymphatic system to occupy distal tissues, where they can hide in immune-privileged sites including extracellular matrix [158]. Evidence suggests that *Borrelia*’s adhesive properties (reviewed below) are key to these protective interactions [195], and its capacity to withstand nutritional challenges in the various environments (considered in Section 5) may also promote prolonged survival at the destination [196]. 

#### Host-*Borrelia* Interactions and Internalization

*Borrelia* has the fundamental capability of interacting with host materials and cells. The flexibility of these interactions is integral to survival in a range of environments. *Borrelia* adhesins, their binding partners and the associated physiological consequences have previously been reviewed [195]. These interactions are complex, with multiple binding partners for each spirochete adhesin and host receptor, as well as highly polymorphic adhesins and variable adhesin expression between strains [197]. In the context of the human, host-microbe interactions facilitate dissemination, colonization and survival, resulting in a highly effective pathogen.

During a tick feeding event, *Borrelia* moves from the tick midgut to the dermis of a mammal. *Borrelia* can colonize the local extracellular matrix (ECM) and traverse the dermis at speeds of a few microns per second [198]. These spirochetes are able to replicate in the dermis and subsequently disseminate both locally and haematologically as the disease progresses from a contained infection to a systemic illness [199]. In vitro co-culture studies have indicated that *Borrelia* interacts with several blood cell types, as well as with endothelial cells (summarized in Table 3). The unique motility by endoflagella and the dissemination and endothelial transmigration of *Borrelia* have been reviewed elsewhere [200]. Investigations into the biomechanics of *Borrelia* vascular interactions have shown that *Borrelia* transfers mechanical load along a series of adhesion complexes resembling selectin-dependent leukocyte rolling as a mechanism of hematological dissemination [201]. In-vivo 3D imaging of fluorescently-labelled spirochetes in mouse models has revealed that spirochetal escape from the vasculature involves a transient endothelial cell interaction followed by dragging and possible stationary adhesion, then transmigration across the endothelial cell layer and escape ‘end-first’ [202]. 

Once disseminated, *Borrelia* can colonize secondary tissue sites throughout the body. In vitro co-culture studies implementing techniques such as adhesion assays, protein interaction experiments and immunofluorescence microscopy, alongside washing steps to remove unbound bacteria, have supported the notion that *Borrelia* adheres to human cell types ranging from chondrocytes to neurons (summarized in Table 3). It has also been reported that *Borrelia* can invade, colonize, and degrade ECM components. The ECM degradation may be attributable to host-derived proteolytic activity through binding to host plasminogen, similar to a hypothesized mechanism of blood-brain-barrier penetration [203,204,205]. On top of host cell adherence and colonization of ECM, *Borrelia* can survive in so-called immune-privileged sites that contain extracellular fluids that do not run through typical lymphatic pathways, such as the eyes, joints, and central nervous system, providing a mechanism for immune evasion. 

Cellular internalization of *Borrelia* has also been proposed as a mechanism of dissemination, immune evasion, host–cell functional damage, and long-term survival [210,219,223]. In vitro co-culture studies providing evidence of adhesion and internalization across a range of host materials and cell types are summarized in Table 3. Several phagocytic cell types have been co-incubated with *Borrelia* leading to bacterial detection and spirochete internalization via a coiling phagocytic mechanism [213,220]. Monocytes and dendritic cells degraded the internalized spirochetes as expected [209,218], whereas in macrophages, there were occasional live *Borrelia* observed within the cell, which were able to be re-cultured [217]. Also of note, the cytokine response following co-culture with monocytes was found to be consistent with that expected for intracellular pathogens [218]. *Borrelia* could be re-cultured after internalization by fibroblasts 28 days post-antibiotic challenge, and there was an observed morphological change, hinting at the relevance of pleomorphic forms in the host environment (reviewed in Section 5.2) [211,212]. After co-incubation with lymphocytes, *Borrelia* was internalized and observed to be motile within vacuoles one to two hours later, and some killing of lymphocytes was observed after one day [180]. *Borrelia* has also been observed to be viable after 20 h co-incubation with neuronal and glial cells following antibiotic challenge [219,220]. When cultured alongside synovial cells, intact spirochetes have been observed at 7 weeks of co-culture and 63 days post-antibiotic challenge [223,224]. 

Understanding the adhesive and intracellular capabilities of *Borrelia* aids our understanding of the survival strategies of this pathogen. More specifically, host–cell interactions have suggested mechanisms of transmission, dissemination, colonization, and immune evasion. Additional research in this area will illuminate pathogenic mechanisms and could identify potential diagnostic and/or therapeutic targets.

## 5. Environmental Challenges and Microbial Adaptations

As described in the preceding sections, the trajectory of Lyme disease is an evolutionary arms race of sorts, influenced by the underlying genetic potential of the pathogen and its capacity to circumvent defenses and exploit vulnerabilities in the host. Research in animal models has demonstrated that the immune response alone is often inadequate to clear infection; hence, prompt antibiotic therapy is recommended. Yet, questions and controversies remain about optimal treatment protocols and the tractability of various LD manifestations to antimicrobial intervention. At the heart of this debate is the question of how *Borrelia* respond to various stressors, such as fluctuations in the biochemical environment and antibiotic exposure, and how lessons learned in vitro translate to the clinic.

Several regulatory pathways allow Bb to sense and react to its surroundings. The stringent response, found in most bacteria, drives global adaptive changes in cell physiology during starvation or nutritional insufficiency. *Borrelia* use the enzyme Rel_Bbu_ to synthesize the common effectors of the pathway, known as alarmones (guanosine tetraphosphate and guanosine pentaphosphate, or (p)ppGpp), which alter transcription by exerting allosteric control over RNA polymerase and accessory proteins to modify their affinities for different promotors (reviewed in [196]). Although basal levels of (p)ppGpp are constitutively produced during borrelial culture, alarmone levels increase during nutrient deprivation to promote Bb survival [225]. Transcriptomic comparison of wildtype and *rel_Bbu_* deletion (Δ*rel_Bbu_*) strains, which cannot produce (p)ppGpp, determined that the stringent response is more diverse during the stationary phase than during exponential growth of Bb in conventional culture, consistent with the role of this pathway in nutritional stress [226]. When acquired by the tick vector, the Δ*rel_Bbu_* mutant Bb failed to sustain population numbers in the tick following its subsequent feed. This decline appears to be reflected in lower rates of transmission to naïve mice, suggesting that the stringent response helps to govern the spirochete populations in ticks as they endure extreme nutrient fluctuation from the unfed to the fed vector environments [225]. Conversely, the consequences of needle-inoculating mice with Bb carrying the same gene deletion seem to depend on the borrelial strain and concentration of inoculum. In one study using strain 297 clone BbAH130, *rel_Bb_* deletion was found to abrogate virulence in mice [227], whereas in another investigation, *rel_Bbu_* mutants on a B31-5A4 background were recovered from distal tissues 5-weeks post-infection [225]. These conflicting observations suggest that the stringent response may not be a universal requirement for the mammalian infection process itself, even though it does appear to be instrumental in borrelial persistence in the tick. The capacity of stringent-defective (Δ*rel_Bbu_*) mutants to establish chronic, disseminated infection of more than 5 weeks duration in mammalian hosts has not been evaluated, although it has been hypothesized that the stringent response may promote survival in nutrient-poor destinations such as collagenous matrices [196]. 

Another signaling pathway of interest in this context is likewise common to a number of bacteria as a mechanism of intercellular communication, to coordinate physiological responses [228]. Quorum sensing in *Borrelia* uses the diffusible pheromone, autoinducer-2 (AI-2), which is generated by the LuxS enzyme via an intermediate (4,5-dihydroxy-2,3-pentanedione; DPD) that undergoes spontaneous rearrangement to form the signalling molecule [229,230]. AI-2 then acts on an unidentified receptor to influence expression of a number of targets, including factor-H binding Erp proteins [231] and VlsE [230], both of which are associated with virulence. Nevertheless, high-resolution profiles of AI-2-associated changes in the transcriptome and proteome have never been reported, so the response remains largely uncharacterized. The contributions of quorum sensing to the enzootic cycle are also somewhat ill-defined. One group reported that a *luxS*-deficient clone of Bb strain 297 retained its infectivity in mice following needle inoculation [232], and also maintained its capacity for tick colonization and transmission [233]. Although the infectivity of the *luxS* mutant strain was confirmed recently by a quantitative approach that evaluated bacterial burden in tissues, a mixed infection with wildtype and mutant Bb favoured the wildtype cells [234]. This result suggests that LuxS may, indeed, contribute to mammalian colonization, dissemination, and persistence, in ways that are not yet clear. 

In other bacterial genera, quorum sensing has been associated with community phenotypes and cooperative behaviours, such as biofilm formation, that impact the progression and drug tractability of disease [228]. These signalling networks have thus become an attractive target for novel therapies to combat recalcitrant infection [235]. Therefore, understanding the intrinsic cellular wiring that underlies borrelial survival strategies and evasive mechanisms, and their nodes of convergence, may prove to be key in pharmaceutical management of the pathogen.

### 5.1. Antibiotics and Borrelia Burgdorferi

Even before the bacterial cause was known for the EM and other Lyme-related symptoms, afflicted patients were being treated with antibiotics (primarily penicillin G) with varying degrees of success [236]. Europeans had been prescribing penicillin for similar tick-acquired rashes as early as the 1950s [237]. The first clinical trial conducted during the late 1970s for LD in North America compared penicillin G, erythromycin, and tetracycline, which represented the predominantly prescribed treatments for LD symptoms [238]. From this trial, penicillin and tetracycline were found to reduce the duration of the EM compared to erythromycin which did not show any significant improvement over an absence of treatment [238]. Once *Borrelia burgdorferi* was identified as the cause of LD, it was possible to test antibiotic susceptibilities in controlled lab experiments, with isolated (in vitro) bacterial cultures and with animal (in vivo) infection models. Such experiments confirmed the results from early clinical trials, that Bb was sensitive to penicillin and tetracycline but indicated that penicillin was not the most effective antibiotic for clearing an infection [239,240,241]. Surprisingly, the latter two studies found cultured *Borrelia* isolates to be quite sensitive to erythromycin but, in agreement with clinical trials, an infection was poorly cleared by this antibiotic in animal models. This finding also highlights the risk in relying solely on in vitro antibiotic susceptibilities to inform clinical outcomes for Bb infections.

#### 5.1.1. Laboratory Determination of Antibiotic Susceptibilities

The relatively unusual biology of the spirochetal pathogen has made it difficult to use internationally-accepted protocols and guidelines that have been established for more common bacterial pathogens. Poor light absorption by *Borrelia* liquid cultures precludes quantifying growth by absorbance, a preferred method for monitoring ongoing culture growth. Inconsistent growth on solid media necessitates alternative methods to measure treated culture viability. Also, the growth media commonly used for Bb is relatively complex and may reduce the antibiotic efficacy (e.g., penicillin incubated in *Borrelia* growth media was reduced to 17% of its initial concentration after 3 days [242]). This lack of easily transferrable guidelines has led to a wide array of experimental conditions to monitor antibiotic susceptibility, including varying definitions used to calculate antibiotic minimum inhibitory concentration (MIC) and minimum bactericidal concentration (MBC) values in the borrelial literature. A 2002 review contained a non-exhaustive list of 12 studies with their different definitions and conditions for determining MIC/MBC values [243]. The earliest (and still widely used) method was to grow Bb cultures in liquid growth media with varying concentrations of antibiotics, and then culture growth was quantified by the inefficient method of visual cell counting using dark-field or phase-contrast microscopy. Multiple variations exist in the literature at each stage for this basic protocol, including (but not limited to): the age of Bb culture before antibiotic addition; the duration of antibiotic treatment; the volume of the liquid culture (i.e., ‘macro’ grown in culture tubes versus ‘micro’ grown in microtiter plates); the temperature and atmosphere (oxygen and carbon dioxide levels) of growth; and, the criteria for cell enumeration (e.g., total number of cells, motile versus non-motile cells, intact spirochetes, etc.). There have been several attempts made in the literature to standardize these types of experiments, but there is little indication of a consensus or an adoption of any particular set of guidelines/conditions. 

The need for high-throughput screening to test thousands of compounds has prompted researchers to develop more efficient assays for measuring antibiotic sensitivities. A fluorescence-based assay that uses a two-dye combination to monitor cellular intactness, and thereby viability, has been optimized for Bb [244]. Another group has optimized a luciferase-based assay to measure ATP levels to monitor viability as a function of energy metabolism [245]. It is hoped that as other groups use these assays, a consensus protocol will be achieved, allowing for the direct comparison of reported antibiotic sensitivities of Bb. However, until high-content techniques are developed for Bb, these high-throughput assays cannot completely replace microscopic analysis of antibiotic-treated Bb. As described in more detail below, Bb can undergo multiple morphology changes in response to antibiotic treatment, and these various morphological forms of Bb can display different antibiotic sensitivities which, in turn, could have clinical relevance.

Even with this high degree of variation in how borrelial in vitro antibiotic sensitivities were measured, some general trends can be observed, as outlined in a 2006 review [246]. Beta-lactam antibiotics had variable effects on Bb: most penicillin-like beta-lactams and third generation cephalosporins showed good growth inhibition, but first generation cephalosporins were poor. For chemical compounds that target the ribosome and protein synthesis, aminoglycosides did not affect Bb but ketolides, macrolides (including erythromycin), and tetracyclines were good in vitro antibiotics. Further complicating the interpretation of antibiotic susceptibility experiments is that different strains have been shown to have different MIC values [247], a common observation for other pathogenic bacterial strains. This strain-dependent variability in antibiotic susceptibility places a lot of importance on clinical isolate strain (or even species) identification, and on having the requisite strains tested with appropriate antibiotics. The age and density of the bacterial culture can also affect the response of the bacteria to antibiotic treatment, with older, stationary cultures generating a higher proportion of cells that persist after treatment than early, log-phase cultures [248]. With many papers devoted to measuring the susceptibilities of *Borrelia* to a wide array of antibiotics, very little has been published on the investigations into possible antibiotic resistance mechanisms, as reviewed in [249].

#### 5.1.2. Resistance: Known Mechanisms and Generation

Common antibiotic resistance mechanisms that are either acquired by horizontal gene transfer or by selective pressure in the clinic have not been described to date for *Borrelia*. Also, few studies investigate the molecular basis of resistance to those antibiotics that show poor activity. Nevertheless, one mechanism that has been studied is the resistance to fosfomycin (or phosphomycin). Fosfomycin normally alkylates an active site Cysteine (Cys) residue in a peptidoglycan biosynthetic enzyme, MurA, which has a naturally occurring Aspartate (Asp) in the homologous position in Bb. When the Bb MurA Asp was substituted with the more common Cys residue, the isolated enzyme was now inhibited by fosfomycin [250]. This modified MurA also had 20-fold higher activity than wild-type Bb MurA, suggesting that Bb exists in nature with peptidoglycan biosynthetic machinery that is not capable of activity levels achieved by most other bacteria. It is likely that the Cys to Asp substitution provides a selective advantage that overcomes the reduction in activity.

Another, more general, resistance mechanism initially identified by bioinformatics was an efflux pump system (BesABC) with sequence homology to the TolC-tripartite multi-drug efflux pumps found in many bacteria [251]. BesC forms an outer membrane pore, and deletion of the *besC* gene has been shown to abolish virulence in a mouse infection model. The deletion strain had higher susceptibility to antibiotics, although the improvements in MIC were modest, in the range of 2–8-fold changes for the antibiotics tested. Naturally occurring antibiotic resistance mechanisms in Bb are poorly understood and under-studied, although some laboratory experiments have demonstrated the potential for generating resistance after antibiotic exposure.

Strains showing increased resistance to coumermycin A1 were generated by spontaneous mutation of Bb grown in the presence of the aminocoumarin antibiotic [252]. The resistance was mapped to a single mutation in *gyrB*, the DNA gyrase B gene and known target of coumermycin A1. In a follow-up study, this coumermycin A1 mutation was transferrable to a sensitive strain via electroporation [253]. Another group selected for erythromycin-resistance in a lab strain of Bb [254], although they were unable to identify any genetic element responsible for the change in resistance. This increased erythromycin-resistance could also be transferred to both Gram+ and Gram− recipients via conjugation, demonstrating the potential for natural dissemination.

The generation of antibiotic-resistant *Borrelia* in laboratory cultures demonstrates that the spontaneous acquisition of resistance can occur. If resistance to treatment were generated in the clinic in a particular patient, it is unlikely that the mechanism could be perpetuated beyond the initial patient [255] in traditional vector-mediated infection wherein the human is considered a dead-end host, although transplacental transmission from mother to fetus could be an exception [256]. Moreover, if resistance was generated in a reservoir species in the wild, the propagation of antibiotic-resistance is much more feasible. The exposure of reservoir species to clinically-relevant antibiotics in nature is improbable; however, that could change. Rodent bait boxes containing doxycycline (and a tick acaricide) have been tested as a means to reduce Bb infections in reservoir animals [257]. In principle, the reduction of *Borrelia* burden in reservoir animals would in turn reduce the probability of spreading this infection to humans. All-in-all though, the potential of selecting resistant Bb strains in reservoir animals makes the use of front-line antibiotics seem ill-conceived and potentially devastating.

#### 5.1.3. Borrelial Antibiotic Persistence 

The ability of Bb to persist after antibiotic treatment can further complicate the choice of treatment for LD in the clinic. In contrast to antimicrobial resistance, the concept of persistence implies the survival, but not replication, of bacteria exposed to normally lethal concentrations of antibiotic [258]. It is a well-documented phenomenon for most pathogens, and has been associated with chronic infection (reviewed in [259]). In laboratory practice, persistence is characterized by a biphasic killing curve in which the bulk of the population is rapidly eliminated, and succeeded by a subpopulation of more tolerant cells with different kill kinetics [260]. The conventional view of bacterial persisters holds that they represent natural phenotypic heterogeneity in a culture, such that at any given time, a subgroup of cells is in a transient state that avoids the mechanism of many antibiotics that rely on metabolically active, dividing cells for their antibacterial function. Persistence thereby renders cells temporarily impervious to an antimicrobial agent [260]. Resistance mechanisms usually arise from genetic alterations that are subject to selective pressures that enrich for the population of resistant cells. Conversely, antibiotic-treated persister cells subcultured in antibiotic-free growth media produce roughly the same proportion of persistent cells when retreated with the antibiotic compared to the first drug exposure [261]. Therefore, persistence is considered a temporary phenotypic exemption from the effects of antimicrobials via dormancy or similar metabolic perturbation, rather than an active countermeasure strategy in response to a drug.

In the case of *Borrelia*, the existence of antibiotic persister cells has been demonstrated by numerous groups, both in vitro and in vivo with animal models and human patients [262,263,264,265,266]. Notably, this persister fraction has been found to tolerate high concentrations of antimicrobials that exceed levels considered clinically achievable [262]. Although the mechanisms of this phenomenon are still largely unknown, there is evidence of both direct (active) and indirect mechanisms of drug avoidance. For example, examining the transcriptome of antibiotic treated cells revealed a shift in gene expression in response to the drugs, including induction of some ORFs associated with stress response. One comparison of doxycycline- and amoxicillin-treated Bb identified targets that were common to both frontline therapeutics, and a number of genes that were differently regulated between the two treatments when dosed at the MBC [248,263]. Although the extended viability, recovery, and regrowth of these remaining cells were not assessed, the distinct gene expression profiles suggest that drug exposure may be a primary trigger of a physiological response, rather than simply enriching for stochastically pre-formed persisters. A subsequent study profiled the transcriptome of cells under doxycycline MBC and during drug-free rebound, and found a pattern speculated to represent onset and resolution of dormancy [267]. Moreover, treated cells were found to retain some infectious capacity in an immunocompetent murine host [267].

Nevertheless, in vitro regrowth experiments that examined cellular dynamics following antimicrobial treatment suggest that the persistence trait is not selected—the population of survivors from the first antibiotic exposure does not fare better in subsequent exposures to the same drug [248,262]. This has also been documented in humans for whom Bb was recovered from the site of an EM both before and after antimicrobial therapy [268]. *Borrelia* isolated from this small cohort of Slovenian patients following treatment did not show an altered susceptibility profile compared to pre-treatment response, despite withstanding drug exposure in the host [268]. This is characteristic of persisters, and not observed with resistant mutants. However, isolates from different patients did demonstrate substantial interstrain variability in MIC and MBC, as mentioned in Section 5.1.1 above. 

As is the case with other bacteria, the persistence phenotype in *Borrelia* appears to be heavily influenced by the physicochemical environment prior to the addition of antimicrobials, arguing against a model in which the drug itself is an exclusive trigger. This is evidenced by drastic differences in the viability profile of a young (exponential) and old (stationary) culture treated with antimicrobials. Several groups have independently demonstrated that borrelial cultures are largely susceptible to doxycycline and other frontline drugs during exponential growth, but become increasingly tolerant as they mature [248,262,269]. Indeed, stationary or late stage cultures are considered persister-enriched, and have become a common laboratory model of treatment-refractory *Borrelia*. This implies that there are priming events or contextual cues prior to drug exposure that render *Borrelia* less vulnerable to a pharmaceutical even before encountering it, which is consistent with the contemporary concept of “triggered persistence” [258]. Mature cultures are presumed to differ from their young counterparts in several ways, including increased cell density, decreased growth rate, accumulation of secreted factors, exhaustion of growth medium, and a build-up of metabolic biproducts. Persister-enriched stationary phase cultures also demonstrate an increased proportion of non-spirochetal morphologies (pleomorphic forms, as reviewed in [270]), which may represent the persistent phenotype, and will be discussed in Section 5.2, below. 

These observations have prompted speculation about the involvement of the borrelial stringent response and quorum sensing in the antibiotic-tolerant phenotype of stationary phase cells. In late stage cultures, ~20% of the transcriptome has been found to be altered under the control of the stringent alarmones, and the corresponding changes associated with this pathway are generally consistent with dormancy-based drug avoidance mechanisms [196,226]. Although deletion of the key stringent response enzyme, *rel_Bbu_*, failed to alter *Borrelia* antibiotic MICs [227], this assay does not directly evaluate the formation of persister cells. Moreover, drug responses of stationary phase Δ*rel_Bbu_* Bb have not yet been tested, so it remains unclear whether the relationship between the stringent response and *Borrelia* antibiotic tolerance is causal or correlational. Meanwhile, there is some indication that eliminating LuxS, the enzyme responsible for biosynthesis of the quorum sensing autoinducer, impacts both borrelial viability and aggregate structures of stationary phase cultures treated with sub-MBC concentrations of doxycycline [271]. Ultimately, clarity is required around the contributions of these and other environmental response networks to *Borrelia* pathophysiology and persistence. Although the stringent response and biofilm formation may seem like complementary stress avoidance mechanisms that converge to generate persisters, this may not be the case in other microorganisms. In *Pseudomonas putida*, for example, induction of the stringent response triggers biofilm dispersal [272], essentially rendering the two strategies mutually exclusive.

Regardless of the underlying biochemical mechanism(s) of persister formation, focus has recently shifted to examining pharmacological strategies for eradication. One such study screened an FDA drug library of 1524 compounds and identified 27 drugs that had better activity on stationary Bb than doxycycline or amoxicillin [273]. The same group later demonstrated that multi-drug cocktails containing daptomycin were more effective than any single agent alone [269]. Another study tested several antibiotics on late-stage Bb cultures but did not find any that could overcome persistence unless the antibiotic was applied with pulse-dosing, a method that allows cells to recover in fresh growth media between multiple exposures to antibiotic [262]. Pulse-dosing was used based on the model that persistent Bb reverts to actively-growing cells during the recovery phase, making them antibiotic-susceptible again. A subsequent study found that pulse-dosing did not eradicate all morphological forms present in a late stage Bb culture, and was inferior to the cocktail approach [274]. Thus, despite intriguing advances, the discipline has yet to identify a consensus strategy, and test its relevance to mammalian disease. The impetus to translate in vitro findings comes from several prongs of investigation that have recovered Bb from mice [275], non-human primates [79], and human Lyme patients [48] after antibiotic treatment. Therapeutic regimens for borreliosis should require additional considerations to ensure the complete eradication of Bb, including this persistent phenotype. 

#### 5.1.4. Clinical Antibiotic Treatment

The first cases of Lyme disease (and EMs prior to the emergence of LD) were originally treated with penicillin G based on success with syphilis, another infection caused by a spirochetal pathogen. As described above, early clinical trials and in vivo animal models demonstrated that penicillin G was not the most efficient antibiotic in either clearing a *Borrelia* infection, or in reducing the duration of symptoms. Current clinical treatment guidelines for many Lyme symptoms recommend doxycycline (from the tetracycline family of antibiotics), amoxicillin (a penicillin-like beta-lactam), or cefuroxime axetil (a beta-lactam-containing second generation cephalosporin), and these recommendations are generally accepted worldwide [57,66]. Doxycycline was first introduced as a treatment in the 1980s [276] based on in vitro susceptibility experiments [239]. Amoxicillin was introduced as a treatment for LD around the same time based on syphilis treatment success [277]. Cefuroxime axetil was tested as a therapeutic agent in the 1990s [278] because of good in vitro Bb susceptibility [279] and fewer complications such as age contraindications and photosensitivity. These recommended treatments have been in use for early Lyme for close to 40 years, but a recent randomized trial in Norway demonstrated their continued efficacy in the treatment of EMs and in preventing the progression of LD symptoms [280]. More serious Lyme symptoms, such as disseminated EMs, neuroborreliosis and Lyme carditis, can also be treated with intravenous ceftriaxone (a third generation cephalosporin), but side effects may be more prevalent [57,66]. Ceftriaxone was originally tested as a Lyme treatment in the late 1980s because it was known to penetrate a variety of tissues well, including the central nervous system, in addition to having a good MIC against Bb. 

Despite the general consensus around the use of doxycycline, amoxicillin, or cefuroxime axetil as frontline agents for treatment of early infection, guidelines differ in the suggested dose and duration of chemotherapy. The IDSA advises ~14–21 days (drug dependent) of oral therapy [66], while this range constitutes the minimum recommendation of the International Lyme and Associated Diseases Society (ILADS), which also emphasizes clinical judgment in determining the optimal treatment protocol [64]. The latter recommendation and provision for extending therapy in early LD is based on “unacceptably high” treatment failure rates, parsed from acute and re-treatment clinical trials, prospective investigations, retrospective studies, and meta-analyses thereof [64]. Estimates of treatment failure, incomplete resolution, sequalae and chronic complications vary widely in the literature, with anywhere from zero [280] to ~40% of participants reporting prolonged symptoms and/or functional impairment [25]. Meanwhile, a recent meta-analysis of randomized antibiotic trials of early localized LD in adults determined that treatment failures were “rare”, although the certainty of evidence for response was considered low [281].

How might these incongruent outcomes be reconciled? Although a detailed critique of clinical trials is beyond the scope of this review, findings from the past decades suggest study design elements that could substantially influence conclusions. These considerations include the geography of participant recruitment, which is associated with borrelial serotype distribution and prevalence, duration of longitudinal tracking, definitions of treatment response, failure, relapse, and syndrome, and the approach to capturing and quantifying subjective symptoms (e.g., fatigue, musculoskeletal pain) in addition to the objective manifestations that are often reported as primary outcomes (e.g., EM resolution). Fundamentally, all trials are constrained by the lack of a definitive indication of “cure”, or microbial eradication, and thus rely on other indications of infection and morbidity that may be subject to interpretation. 

As a case in point, three studies that were intentionally developed to evaluate post-treatment LD sequelae arrived at vastly different estimates of protracted disease burden, despite enrolling participants with EM lesions and following their response to the same antibiotic. When Cerar and colleagues recruited 285 patients with localized LD from Slovenia, treated with 15 days of doxycycline or cefuroxime axetil, and evaluated subjective symptoms experienced by the patient cohorts and a non-LD control group at 6 months (exam, interview) and 12 months (exam, interview, questionnaire), they concluded that the frequency of new or increased symptoms in the LD groups did not differ significantly from that of the control cohort [282]. Meanwhile, Aucott’s team enrolled 63 American patients exhibiting EM and symptoms of illness, and evaluated the cohort over a 6-month period, including a 3-week intervention with doxycycline during acute disease. Exams, interviews, and validated questionnaires assessing functional impact (SF-36) and mental health (Beck Depression Inventory) were administered at each of 5 visits, leading to the discovery that 35% of participants fulfilled general criteria for PTLDS at 6 months [25]. When the same group applied an operationalized definition of PTLDS (described in Section 2.2) to a patient cohort (n = 74) and controls in a subsequent study, only 11% of participants met the case definition, while 28% experienced either ongoing symptoms or functional impact but not both, thereby disqualifying them from the diagnosis [54]. The latter comparison illustrates the pivotal role of definitions in determining study outcomes and ultimately influencing recommendations, which in turn emphasizes the need for consensus language that also represents biologically- and clinically-relevant phenomena. 

Beyond terminology, the aforementioned European and American studies differ in important ways, including the disease status of the patients at the time of enrollment, and the identity of the pathogen. Whereas Cerar et al. excluded participants with multiple EM and followed a cohort with fewer than two symptoms on average at baseline [282], Aucott et al. recruited patients with evidence of early systemic disease (mean ~10 symptoms) in addition to EM, who then received a longer course of doxycycline [25]. Although the pathogens were not recovered or genotyped from American patients, geography predicts that they were most likely *B. burgdorferi* s.s., whereas *B. afzelii* was by far the most prevalent isolate identified in the Slovenian patients (86.9% of cultivable organisms) [282]. This is a highly relevant distinction considering the discussion in Section 4.1 that borrelial genospecies and serotype influence host invasion and virulence. Notably, early manifestations of European *B. afzelii* are less severe than North American LD [125], and indeed even *B. burgdorferi* in Slovenia was found to be less aggressive [126], possibly due to the scarcity of the more virulent OspC type A [150]. Incongruous estimates of post-treatment disease burden arising from these studies could reasonably reflect genetically-distinct populations of *Borrelia* and LD that vary by virulence and geography. It is important to note that despite the disability they documented in their PTLDS cohort, Aucott’s group did not evaluate patients for microbiological evidence of ongoing infection.

With these considerations and findings in mind, it appears that first-line antimicrobials may not be universally effective at preventing protracted complications, although it remains unclear whether individual drugs differ in their influence on long-term quality of life. The findings also suggest that there may be an unmet need for novel therapeutics early in disease, particularly in regions with a high prevalence of invasive serotypes where patients may be at increased risk of disseminated, treatment-recalcitrant disease. This speculation is likewise spurred by in vitro work, discussed in Section 5.1.3, that suggests that Bb strains can differ in their MICs, and that antibiotics may instigate persistent bacterial phenotypes. Ideally a font-line therapeutic agent should not push surviving cells toward a treatment-intractable state, and as described above, some research is now focusing on the relevance of the transcriptomic changes that occur in *Borrelia* in response to conventional antibiotic treatment [267].

For LD patients who do experience prolonged, relapsing, or new-onset symptoms despite receiving recommended treatment, the question of appropriate intervention strategy is the subject of controversy and uncertainty. In the United States, several randomized, placebo-controlled clinical re-treatment trials were conducted to determine whether IV ceftriaxone with or without adjunctive doxycycline could resolve persistent manifestations, particularly fatigue and neurocognitive impairment [283,284,285,286]. Two of the four studies identified improvements in some domains of interest, but ultimately recommended against the broad adoption of the protocol due to IV-related complications [284,286]. 

Nevertheless, findings have been misrepresented in some subsequent literature [287], and the lack of robust recovery and protocol endorsement has in some cases been erroneously interpreted as evidence that persistent symptoms do not have an infectious etiology. Such studies can only draw conclusions about the dose and duration of the drug under investigation, as it relates to the specific outcome measures established for the particular population being evaluated. Indeed, trial success would depend on additional conditions being met: (i) if present, the residual pathogens must be tractable to the antimicrobial of choice, for which (ii) dose and duration have been established in an evidence-based manner, and (iii) inclusion criteria should distinguish participants who are good candidates for the particular therapy. As discussed in Section 2, laboratory tests of active infection have yet to be validated and broadly adopted, and there are several hypotheses of treatment-refractory illness including immune dysfunction, antigenic debris, and host tissue damage, for which antibiotics would not be appropriate. The possibility that the treatment-refractory population is heterogeneous and may not respond uniformly to intervention was further suggested by a recent registry-mining study that evaluated responses collected from American patients by an NGO. The analysis of MyLymeData identified 34% of nearly 4000 registrants as “high responders” to antibiotic therapy [288]. The distinguishing features of the cohort are yet unknown, and although there are many variables inherent to this study design, response diversity emphasizes questions of treatment tractability.

To that end, the aforementioned American RCTs for longstanding symptoms were published between 2001 and 2008, whereas laboratory modelling of persistent *Borrelia* and evaluation of its drug susceptibilities is still emerging, as described in Section 5.1.3. Notably, recent in vitro work suggest that go-to LD drugs, including ceftriaxone, have markedly reduced efficacy against persistent forms of the bacterium [269,274]. Consequently, novel treatments specifically targeting persistent Bb infections are beginning to be used in the clinic. Dapsone, a drug used for dermatological pathologies such as acne and leprosy, has shown some efficacy for treating persistent Lyme symptoms in a preliminary clinical trial [289]. An anti-alcoholism drug, disulfiram, demonstrated positive outcomes for three patients with relapsing neuroborreliosis symptoms who were monitored on the drug after conventional treatment failed to adequately control their disease [290], and a clinical trial has been initiated to study the safety of disulfiram treatment for post-treatment LD symptoms (clinicaltrials.gov, NCT 03891667).

One aspect of clinical treatments specific to Bb infections that is under-studied is the pharmacokinetic and pharmacodynamic properties of the various antibiotics. These properties have been tested in model organisms (e.g., non-human primates [79]) but very rarely in Lyme patients. Another poorly understood aspect of Bb treatments is the observation that, for a subset of patients, symptoms can worsen after antibiotic treatment. This apparent paradox has been termed the Jarisch–Herxheimer Reaction (JHR) and has been observed in other spirochetal infections, such as syphilis and leptospirosis (as reviewed in [291]). For relapsing fever, a tick-borne illness caused by the spirochete *Borrelia recurrentis*, the JHR symptoms that occur after penicillin treatment are preceded by an increase in blood serum levels of cytokine TNF-alpha and followed by increases in IL-6 then IL-8 [292]. A small controlled trial of 40 relapsing fever patients was conducted in which one half of the patient group received antibody fragments specific to TNF-alpha in order to prevent TNF-alpha activity [293]. Patients who received the TNF-alpha antibodies showed a suppression of JHR symptoms, but not a complete elimination of JHR, indicating that the treatment was not fully effective. Others have suggested that there may be a treatment-inaccessible sub-population of TNF-alpha that causes the JHR symptoms, or that other mediators exist [294]. The actual molecular component of Bb that initiates JHR in Lyme patients is currently unknown, and the reaction in Lyme patients is usually reported in the literature as being mild relative to other infections. One conceivable cause of increased symptoms and serum cytokine levels post-treatment is the release of immunologically reactive fragments generated by antibiotic-induced cellular destruction [295], including peptidoglycan [296,297]. Understanding the root cause of the JHR would lead to treatments that reduce or eliminate these episodes. 

The clinical picture may be further complicated by co-morbidities that resemble, or exacerbate LD. Any additional infection (bacterial, viral, parasitic, etc.) acquired either before, during, or after the initial Lyme infection can confound both diagnosis and treatment of LD and may even affect the progression of LD symptoms. There is an immense number of potential combinations of co-infections, but a general lack of peer-reviewed literature investigating the trajectories of such polymicrobial infections. The majority of the available literature on co-infections has focused on the diversity and epidemiology of tick-borne pathogens present in the vector and the various mammalian hosts, including human patients. Two recent examples collated the available data for two of the most common infections that coincide with LD, the parasite *Babesia microti* that causes Babesiosis [298] and the bacterium *Anaplasma phagocytophilum* (formerly known as *Ehrlichia phagocytophilum*) that causes Anaplasmosis. [299]. How multiple infections co-exist in a human host is still poorly understood, including the possible effects of co-infections on symptoms and disease progression. What is clear is that better diagnostics for LD (and other infections) will help to determine if symptoms are caused solely by Bb or by a polymicrobial infection. This, in turn, will allow for treatments to be tailored to the specific infection(s).

#### 5.1.5. Alternative Anti-Microbial Treatments 

New antimicrobial treatments are being sought to improve clinical outcomes and provide treatment options when recommended treatments are ineffective or contraindicated. Several, more ‘natural’, compounds have been tested, including essential oils [300], phytochemicals [301] and bee venom [302]; however, the financial input required to take these compounds to clinical trials makes it unlikely that these treatments will get the rigorous testing they would require to be clinically accepted. A number of studies have screened FDA-approved drugs for their potential as novel Bb antibiotic candidates [245,273], and have identified antibiotics previously untested on Bb that showed good efficacy. Studying drugs that are already FDA-approved bypasses the requirement for the early stages of clinical trials, and reduces the financial burden in bringing new Bb treatments to the clinic. Additionally, drug combinations (as many as four) have been tested to improve the elimination of Bb infections, including the reduction of persistent Bb [269]. Nevertheless, a concern with using multiple antibiotic cocktails to treat a single infection is that the aggressive and untargeted strategy could lead to a more global elimination of the patient’s microbiota making the patient more prone to subsequent infections, such as *Clostridium difficile*.

To generate more *Borrelia*-specific treatments, some research has identified potential new targets for antimicrobials. Glycosaminoglycan (GAG) binding proteins have been proposed to be a target to reduce virulence by preventing the attachment of Bb to host tissues [303] as has been successfully shown for several other Gram- and Gram+ bacteria (reviewed in [304]). Pre-treatment of an animal host with dalteparin (a GAG) or pre-infection exposure of Bb to dextran (a GAG analogue) significantly reduced the interactions of Bb with the vasculature of a mouse host [305]. Another study has investigated methylthioadenosine/S-adenosylhomocysteine (MTA/SAH) nucleosidases, proteins involved in purine and methionine salvage pathways, as a new Bb antibiotic target [306]. The importance of MTA/SAH nucleosidases to Bb was supported by the presence of three copies of these nucleosidases encoded in the Bb genome, whereas all other bacteria appear to encode a single copy. Inhibitors of MTA/SAH nucleosidases were shown to be very effective at inhibiting Bb growth in vitro [306], establishing these nucleosidases as good targets to investigate further.

### 5.2. Pleomorphy

A strict definition of pleomorphism is “the quality or state of having or assuming various forms”. This term is synonymous with polymorphism, and is a well-documented attribute of several types of bacterial species that typically refers to their capacity to alter cellular shape [307]. Examples of morphological plasticity in bacteria have led researchers to hypothesize that this feature likely serves a role in reproduction and survival under stressful conditions [308]. Reviews of this widely conserved feature can be found elsewhere [307,309,310]. Likewise, members of the phylum Spirochaetae are not exempt from this trait, and notable examples include *Treponema* spp. [311,312], and *Borrelia* spp. [270,313,314,315]. Indeed, a report in the 1990s of atypical borrelial morphologies recovered from human specimens spurred interest in the phenotypic plasticity of this organism [316]. In light of these findings, it is speculated that pleomorphy plays a role in the pathology of LD and that these diverse morphological forms may confer, or contribute to, improved persistence within the human host [270,313,314,317,318]. There are many studies that demonstrate the in vitro pleomorphic capabilities of *Borrelia burgdorferi* [313,314,315,319,320,321], and a few reports that have identified similar forms in clinical samples [313,316,322]. Pleomorphism can encompass a variety of morphologies, so it is important to develop consistent terminology when referring to these forms. Several non-spirochetal forms have been described for *Borrelia* spp. (summarized in Table 4).

The collective phenotype of homo- or heterogeneous cultures can also change through the formation of aggregated bacterial communities, or biofilms. Although these may not fall under a strict definition of pleomorphism, which tends to focus on the individual cell, they have likewise been implicated in disease, and we include them in briefly in this discussion for illustrative and comparative purposes. Borrelial clusters are routinely observed in cultures of type strains, and community structures have also been reported histologically in human biopsy and autopsy specimens [266,323]. Microbiologically, biofilms are defined as a structure or matrix composed of extracellular polymeric substances (EPS), housing a host of microbial cells, that adheres to a static surface (biotic or abiotic) [324].

The terms identified in Table 4 provide a framework for a general understanding of the pleomorphic forms of *Borrelia*. However, limitations in ultra-structural, functional, and inductive data hinder a more sophisticated comprehension of these morphologies. As a result, several of these terms have been used interchangeably in the literature, reducing clarity and consistency, and occluding interpretations of observations beyond speculation. Here, we consolidate findings from the literature specifically concerning the induction and characterization of round-bodied (RB) pleomorphic forms of LD-causing *Borrelia* in order to highlight what is known, and areas that require further investigation, reconciliation, and validation necessary to advance the field and to improve our understanding of their role in disease.

**Figure 4:**
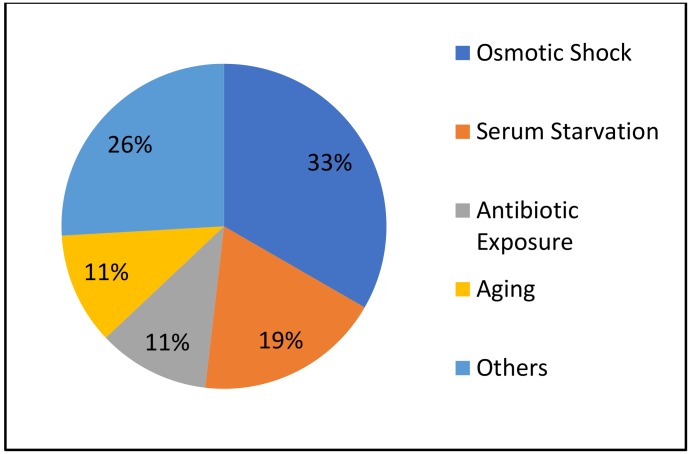
Peer-reviewed publications documenting in vitro conversion of *Borrelia burgdorferi* into round bodies (RB), compared by induction method.

As seen in Figure 4, the most commonly published method of inducing round RB forms of *Borrelia* spp. is through osmotic shock via exposure to distilled water [313,314,321,327,328,329]. 

Water shock is also consistently reported to be the fastest for the conversion of large proportions of spirochetes into round bodies (Table A1). Efforts to determine the viability of RBs largely center on whether they are able to revert to regular, reproductive spirochetes when re-introduced into standard growth medium conditions (Table A1). There are several instances in which researchers appeared to be successful in culturing live, motile spirochetes from water-shocked *Borrelia* (Table A1). A potential flaw with this technique is the question of whether the small proportion of spirochetes that did not transform into round bodies (bystanders) are able to grow and divide when re-introduced to BSK. The subsequent replication of these bystander cells may falsely be attributed to the reversion of round bodies into motile, dividing spirochetes. Filtration has been used to separate round bodies from helical forms so that the activity of the round bodies and flat-wave spirochetes can be assessed directly [285,300]. In such instances, the authors report that filtering out round bodies in parallel treated cultures has yielded cultures of flat-wave spirochetes (bystanders) that show no sign of growth over time, thereby affirming that bystanders are not viable and that the observed growth must come from viable, reverted round bodies [285]. The authors went on to confirm the growth of reverted round bodies in cultures treated for 10 min, 2 h, and 4 h, but not from longer exposure periods [285]. This finding is supported by the observations of others, although not all of these studies accounted for bystanders [284,299,300]. Monitoring metabolic activity, including measuring ATP and RNA levels, has also been used to infer the viability of round bodies. Round bodies can be labeled with an RNA-specific stain [301] and they also exhibit ATP synthesis, albeit at a lower level than spirochetal cells [285]. The osmotic conditions and maximum duration of exposure before failing to revert back into growing spirochetes does not present a convincing argument for a direct role in persistent human infection; however, understanding the molecular mechanisms behind these transformations may help to further elucidate *Borrelia*’s tolerance for stress.

**Figure 5:**
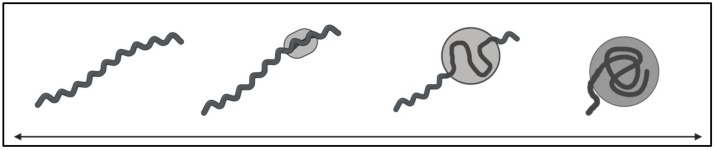
Proposed stages of transformation of *Borrelia burgdorferi* to and from round-bodied (RB) forms.

Another, relatively common, means of inducing round-bodied forms of Bb is nutrient deprivation, particularly serum starvation. *Borrelia* spp. are known to lack the necessary requirements for de novo synthesis of amino acids and, as a consequence, are obligate parasites, harvesting amino acids from their tick and mammalian hosts [330,331]. As such, *Borrelia* spp. may be sensitive to the abundance of host-derived nutrients in their immediate environment. Exposing Bb cultures to a mammalian cell culture medium (RPMI-1640) that lacks animal serum and bovine serum albumin (BSA) (common, possibly essential, components of *Borrelia* growth media) is one method for monitoring the effects of serum starvation. The consistency of RPMI-induced pleomorphic forms is questionable as round bodies were described to have formed in cultures at drastically different rates by different authors (Table A1). Despite the characterization done by Alban et al. [319], these findings cast doubt over the reproducibility of this technique [285]. Given *Borrelia’s* inability to synthesize crucial amino acids [11], serum starvation is a critical concern for the survival of the spirochete and has been linked to *Borrelia*’s stringent response [225,332]. Likewise, characterization of *Borrelia’s* stringent response may suggest a role for pleomorphism as a downstream response to alarmone signalling and metabolic dormancy for surviving nutritional stress [304,305]. However, deletion of a key gene involved in the Bb stringent response, *rel_Bbu_*, was found to decrease cell survival, and increase the number of round body forms under starvation conditions [225]. This suggests that RBs are not unique or exclusive signatures of the stringent response, nor are they always viable. It also remains to be seen whether Bb responds to immune and antibiotic challenges in a similar fashion to nutrient deprivation. Paradoxically, exposure to human serum has been used as another means of inducing pleomorphic forms [314], thereby highlighting the need for further investigation into whether various induction techniques truly produce the same morphotype. Comparisons of osmotic shock- and serum starvation-induced pleomorphic forms, using TEM data, suggest a similar mechanism of transformation into round bodies: (1) expansion of the outer membrane followed by (2) whirling of the protoplasmic cylinder within (Figure 5). These observations suggest that a common mechanism of stress response is activated in either condition, leading authors to infer that Bb is capable of an encystment-like survival strategy.

Antibiotic challenge has also been used to induce pleomorphic forms of *Borrelia* (Figure 4; Table A1). Penicillin, amoxicillin, doxycycline, and ceftriaxone have all been reported to induce spherical forms [333,334]. In the case of penicillin and ceftriaxone (both beta-lactam containing antibiotics), the induced morphology resembled water-shocked round bodies in that they were both described as having convoluted spirochetal structures within a spherical body [333]. The authors were unable to determine whether these forms could survive past 96 h of incubation with the antibiotics, or revert into a motile, helical form [333]. In contrast, doxycycline was reported to induce “multiple ovoid structures,” of unknown function [333]. The rates of onset of these atypical forms are difficult to compare given the paucity of examples within the literature concerning *Borrelia*. Amoxicillin, another beta-lactam, was reported to cause a significant conversion of over 96% of the spirochetal sample to round-bodied forms after 3 days of incubation [334]. Although it may be difficult to compare across different types of antibiotics, amoxicillin and penicillin both share the same mechanism of action through binding penicillin-binding proteins (PBPs) in the bacterial periplasmic space to inhibit the synthesis of the peptidoglycan wall. As such, it is interesting to find that penicillin, at a concentration of 4.0 μg/mL, would induce irreversible transformation of spherical forms after 96 h of incubation whereas amoxicillin, at a concentration of 50 μg/mL, would yield reversible round bodies after 72 h of incubation time (Table A1). Although the difference in incubation time is significant, the greater than 10-fold difference in antibiotic concentration is noteworthy. An earlier study found the MIC90, where 90% of the bacteria are inhibited, of amoxicillin to be less than 0.03 μg/mL for *B. burgdorferi* [335]. 

Pleomorphic forms of *Borrelia* spp. are not only inducible in vitro, but they have also been detected within the enzootic life cycle of the spirochete, specifically within the midgut of the tick vector [336]. These forms have been loosely described as spherules, granules, gemmae, cysts, coccoid forms, and round bodies at different points throughout the literature (Table 4; [270,313,314,328,329,333,334,337]). Likewise, a wide variety of methods have been used to generate and evaluate these forms, and yet the resulting morphotypes are treated synonymously (Table A1). Whether these techniques and terms all describe the same phenomenon in an “all roads lead to Rome,” style of observation has yet to be determined. Differences in conversion rate, average dimensions, and viability between these induction methods demand caution over how we treat these various forms and the terminology we assign. It has been argued that part of the problem in understanding pleomorphic forms of Bb is the confusing application of terms such as “cyst/cystic” and the ambiguity of “round bodies” [338]. Likewise, whether these induced forms are identical to, or represent a heterogeneous population with, forms identified in clinical samples with persistent infection remains to be fully elucidated [313]. As such, there exists a need for further characterization of these forms in the context of multiple induction methods, defining structural changes, and survival mechanisms in order to properly assess any hypothesis about their role in disease and persistence within the human host. 

As mentioned above, *Borrelia* have also been observed in clusters and aggregates of various sizes in vitro [325,326,339,340] and in vivo [323,341]. These clusters are capable of forming on a number of biotic and abiotic surfaces [325], and have been reported to contain extracellular DNA (eDNA), alginate, and calcium— all known components of the extra-polymeric substance (EPS) matrix characteristic of bacterial biofilms [325,340,342]. Consequently, these findings have raised the question as to whether biofilm formation is a mechanism by which the Lyme spirochetes may persist within treated hosts, as the antibiotic-resistant features of bacterial biofilms are already recognized and documented in other pathogenic species [343,344]. *Borrelia* biofilms display greater in vitro tolerance for commonly prescribed Lyme antibiotics [345], and may potentially confer more severe symptoms than their planktonic counterparts, as demonstrated in a mouse model [326]. A recent study has identified polymicrobial biofilms featuring *Borrelia* spp. and *Chlamydia* spp. in human tissue [341]. Interestingly, the high presence of round bodies is closely associated with biofilm formation [325], although the exact relationship between these morphological variants has not been well studied.

Pleomorphism and persistence have often been cited as intertwined features in other bacteria, including pathogenic *E. coli* and *Treponema pallidum* [313,346]. A recent study provided strong evidence for the role of pleomorphism in urinary tract infection *E. coli,* in which cell wall-deficient L-forms were reported to confer greater tolerance to penicillin [346]. However, for Lyme *Borrelia,* the relationship between pleomorphism and antibiotic susceptibility is less established. While in vitro evidence for *Borrelia’s* pleomorphic capabilities exists within the peer-reviewed literature, the role of these forms in the diagnosis, progression and treatment of LD remains controversial. It has been noted that there is a paucity of documented RB pleomorphy in cases of persistent infection, chronic disease, and/or post-treatment LD syndrome, and the interpretations of existing findings have been questioned [338]. This discrepancy could be explained by several issues, the first of which pertains to the definition of PTLDS or chronic Lyme. As discussed in Section 2.2 and Section 5.1.4, the current case definitions and conventional interrogations of PTLDS have de-emphasized the potential contribution of persistent infection to ongoing illness, so there appears to have been little impetus to evaluate microbiological correlates of disease [314]. Secondly, cultivation of pleomorphic forms using BSK-based culture media may promote the reversion to planktonic, flat-wave forms. If metabolic dormancy and the stringent response are, indeed, intimately associated with round body formation, the re-introduction to non-strenuous or nutrient-rich BSK may mask the presence of pleomorphic forms when viable bacteria do become detectable. Thirdly, a fundamental lag in the understanding of the molecular underpinnings of pleomorphic forms has resulted in the inability to definitively differentiate viable aberrant morphotypes from debris and non-specific or artifactual staining using immunohistochemical methods. Likewise, traditional micrograph analysis protocols may be optimized to detect classical spirochetal shapes, at the expense of other phenotypes. Therefore, it is possible, even probable, that pleomorphic forms of *Borrelia* are involved in the persistence of the pathogen within the human host, given the abundance of data that reflects *Borrelia’s* pleomorphic capabilities as well as the precedence set by other pathogenic spirochetes (*T. pallidum*), but a lack of fundamental microbiological/molecular characterization of these alternate morphotypes has hindered our ability to reliably interrogate clinical samples for their presence.

## 6. Conclusions and Future Frontiers

Integrating findings that have been generated over decades from many different subspecialties portrays Lyme disease as a complex biological, medical, and socio-political scourge. Elements of the field that previously appeared irreconcilable have begun to resolve as the research enterprise expands and adopts more sophisticated techniques that increase the sensitivity and throughput of analyses. Yet, major gaps still exist that need to be addressed in order to improve outcomes. High-priority issues raised by patients and clinicians—diagnostic uncertainties and intervention protocols for recalcitrant illness chief among them—can be evaluated from the perspectives of fundamental microbiology, host-pathogen biology, and human physiology, although biomedical research itself has been constrained by the lack of objective, relevant, biological markers and metrics of disease. Box 1 consolidates some of the major themes and recommendations explored in this review. Across the board, there is a recognized need for diagnostic tools that capture all stages of the disease, and can be used to monitor the infection status and evaluate microbiological eradication [347]. As high-quality direct-detection techniques evolve from interdisciplinary research, they will in turn accelerate clinical studies by affording investigators a higher resolution biological view of disease progression and treatment outcomes.

Box 1 **Summary of Major Conclusions and Implications.**

Clinical signs and two-tiered serology consistent with U.S. CDC recommendations are the most common approaches used in diagnosis and research study inclusion criteria. (Section 2.1)
Often do not encompass the entirety of LD cases and could lead to under-diagnosis, as well as bias in research study design.Development and validation of direct, high-sensitivity Bb detection methods will provide a basis for standardization of study inclusion, and disease monitoring LD presentation has been classified by stage of infection (early localized to late disease) and also by outcome (treatment refractory Lyme arthritis (TRLA), post-treatment Lyme disease syndrome (PTLDS), chronic Lyme disease (CLD)). (Section 2.2 and Section 2.3)
Latter terminology has been used inconsistently in literatureCLD is under-represented in research in part because of lack of consensus criteriaMechanisms of protracted disease are disputedHost genetic predisposition to treatment-refractory LA has been proposed based on an autoimmune mechanism associated with HLA-DR alleles. (Section 3.1)
Does not appear to account for other persistent manifestations of LDTRLA has also been linked to specific serotypes of infecting Bb (RST 1)Host diet and blood cholesterol levels could be implicated in the disease progression. (Section 3.2)
Hypercholesteremia is associated with higher spirochetal load in the blood and joints of the infected animalThe role of eicosanoids in disease progression requires further investigation, as well as the ability of NSAIDs to aid in the treatment of diseaseThe invasive potential of Bb is associated with serotype. (Section 4.1)
Mechanistic determinants of serotype-associated phenotype are largely uncharacterizedLong-term implications (PTLDS, CLD) of infection by different serotypes are unknownMammalian host colonization requires an arsenal of strategies to move through the body, evade and subvert the immune system, and invade distal tissues. (Section 4.2)
Produces sustained infection in reservoir species that maintains enzootic cycleLongstanding infection also documented in human patients; more challenging to studySignalling pathways governing Bb population dynamics are incompletely characterized. (Section 5)
Stringent response and quorum sensing may influence virulence and persistence, and could represent druggable targetsBb antibiotic resistance and persistence have been described in vitro. (Section 5.1)
Persister cells are speculated to participate in treatment-resistant diseaseUnclear whether laboratory model of persistence aligns with Bb adaptations and survival in hostDoxycycline, amoxicillin, or cefuroxime axetil are first-line agents for treatment of early infection in North America. (Section 5.1.4)
Long-term outcomes following acute intervention vary (0 – ~40% morbidity, depending on study geography and design criteria)Conventional LD drugs demonstrate poor activity against lab-modelled persistent BbNo consensus on therapeutic strategy for treatment-resistant manifestationsBb is capable of adopting pleomorphic forms under stressful conditions, in vitro. Morphologically similar forms have been identified in tissue samples from patients with confirmed infections with *Borrelia*. (Section 5.2)
It is unclear whether different inductions of pleomorphic forms are homogeneous or heterogeneous in their mechanism of transformation, ability to survive harsh environments, and viabilityIt is unclear whether induced pleomorphic forms are identical to pleomorphic forms identified in clinical samplesFurther characterization and comparison of laboratory-induced versus clinical forms is required

### 6.1. Classifying and Studying Lyme Disease

Fundamentally, the discipline has struggled to define and investigate the breadth of manifestations that have been attributed to Lyme disease. Primarily, this represents intentional investment in well-defined case architecture to the exclusion of what was not long ago derided as hysteria and fringe science [348]. However, with the escalating incidence of LD and growing acknowledgement of complications, there is increasing interest in, and concern around, presentations that fail to conform to conventional expectations. Indeed, the serological definition of the disease, once considered almost infallible by the establishment, suffers from well-documented technical limitations (e.g., delays due to humoral immune response development), in addition to growing evidence of *Borrelia* infections that remain seronegative. The potential for considerable underlying heterogeneity in the human LD population, as determined by factors such as invading serotype, comorbidities, polymicrobial infection, host seroconversion status, genetic and environmental susceptibilities, treatment regimen, etc., suggests that subgroup analysis can be very informative, and that caution should be exercised when making inferences from any given cohort to the broader population.

When considering protracted disease classifications, it is pertinent to note that terminology has not been used consistently in the literature, clinical practice, or socially. Post-treatment (PTLDS) and chronic Lyme (CLD) may also be challenging to delineate due a high degree of variability between patients, and the lack of responsive biological correlates indicative of disease progression and resolution. Based on consensus definitions, however, it is possible to distinguish PTLDS and treatment-refractory Lyme arthritis (TRLA), as the terms describe mutually-exclusive, longstanding manifestations. PTLDS requires the resolution of objective disease presentation, whereas TRLA specifically focuses on objective, relapsing, or non-resolving joint effusion. The genetic predisposition to OspA-stimulated autoimmunity that has been hypothesized to promote development of TRLA was not found to factor in the clinical trajectory of CLD, according to one study that probed this association (Section 3). While TRLA is estimated to occur in only a small percentage of cases [349], >10% of early LD diagnosed on the American Eastern seaboard progresses despite timely intervention to fulfil operationalized criteria for PTLDS, while another ~30% of patients experience ongoing symptoms or impairment, but fail to meet the definition [54] (Section 2 and Section 5). Overall then, the postulated heritable autoimmune predisposition of the host accounts for only a minority of LD sequalae, while the rest remain largely uninvestigated. 

Meanwhile, the variability in presentation and lack of consensus criteria around CLD have often excluded this population from high-quality research investigation. Some sources suggest that this umbrella term captures some of the more debilitated patients [350]. Yet, with the exception of case studies and registry-mining initiatives, which are generally downgraded in the hierarchy of medical evidence, biomedical research is largely failing to adequately represent this cohort. The most common explanation for dismissing the CLD population is a lack of objective evidence of prior or ongoing *Borrelia* infection, and indeed, debates continue about whether chronic “alternatively diagnosed” Lyme is in fact chronic fatigue syndrome (CFS) [72]. But what is CFS? Like CLD, CFS/myalgic encephalomyelitis has thus far defied a unified physicochemical definition, and endured decades of scorn and marginalization in the medical community instead of receiving an adequate infusion of resources to elucidate the underlying pathology. Shuffling ill patients between poorly-defined “syndrome” classifications without addressing fundamental pathophysiological mechanism and improving outcomes is counterproductive. A paradigm shift is required to enable appropriate and relevant evaluation of such patients without a priori assumptions. 

### 6.2. Clinical Microbiology, Pathogenesis, and Treatment

It has become increasingly apparent that complex interactions between *Borrelia* and host materials, including cells and extracellular matrix, play an important role in transmission, immune evasion, dissemination, colonization, collateral damage, and long-term survival within the host, and are integral to the spirochete’s success as a pathogen. The stable of known virulence factors therefore includes a number of adhesins that mediate host-pathogen associations. The various immune subversion strategies employed by *Borrelia* provide additional impetus to move away from diagnostic techniques and disease definitions that depend on host immune response to infer infection. The capacity to divest from a 30-year old paradigm will require the confluence of host biology, microbiology, and technology.

A major finding of the past several decades is that human invasiveness and pathogenicity vary across Bb strains, which, in turn, can be grouped according to genetic classification schemes. The typing system based on OspC alleles (serotype) has proven to be informative in distinguishing strains that remain localized to the tick bite site from those that disseminate throughout the body, and in correlating strains to biochemical characteristics of early illness. While an association has also been shown between RST 1 (OspC type A and B) and treatment-refractory LA, serotype-associated investigations of CLD/PTLDS have not been undertaken to our knowledge. Although the underlying mechanistic determinants that define the divergent phenotypic properties of the serotypes remain to be fully elucidated, laboratory mouse modeling suggests that simple phylogenetic analyses do not tell the whole story [351]. The virulence and invasiveness of a strain depend not only on the fundamental blueprint (genome) of the pathogen, but also on the way in which those instructions are mobilized (transcriptome, proteome). 

**Figure 6:**
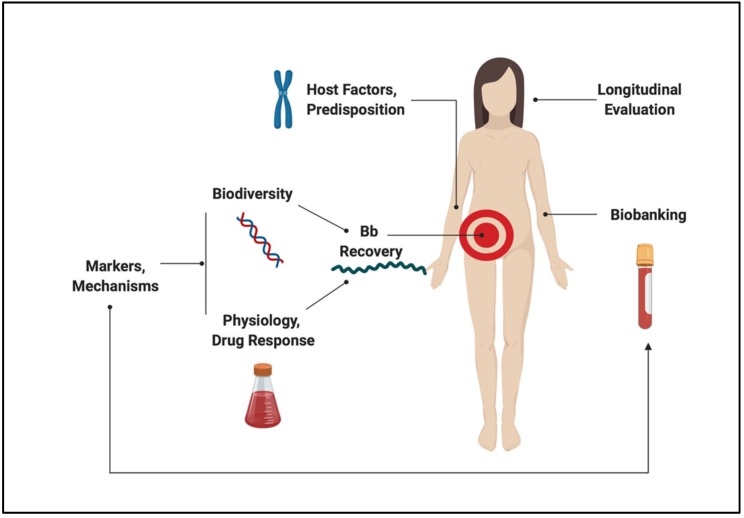
Hypothetical workflow demonstrating the integration of longitudinal clinical assessments, improved microbiological recovery and characterization, and iterative re-evaluation of archived specimens.

Indeed, increasing both the genomic and proteomic resolution of strains and relating them to human disease trajectory should be a priority (Figure 6). Such efforts are nevertheless constrained by low recovery rates of *Borrelia* from human clinical specimens, thereby necessitating improved isolation strategies and/or culture-free characterization platforms that can parse strain-level “omic” detail in situ from complex starting material. Mosel et al. demonstrated the utility of a targeted strategy using multilocus PCR coupled to mass spectrometry to genotype Bb directly from human blood [147], and whole *Borrelia* genomes have also been assembled directly from ticks [352].

Although bypassing culture reduces bias in clinical association studies, pathogen recovery is still required to fuel in vitro analyses. To date, the majority of laboratory investigations of *Borrelia* have revolved around a handful of reference strains (B31 and N40, both *I. scapularis* derived, being the most prevalent [351]). Concerns around laboratory adaptation and potential plasmid loss aside, there is a troubling lack of borrelial biodiversity represented in fundamental research. Discoveries of Bb heterogeneity in nature and the clinic are not yet reflected in vitro, raising questions about the medical applicability of laboratory manipulations. As mentioned in Section 5.1.1, strains are known to differ in their antibiotic MIC values, although the extent to which serotype influences drug tractability remains to be fully investigated. Improving clinical recovery will generate a more diverse repertoire of strains on which to base in vitro work, and although isolates propagated under artificial conditions cannot be considered host-adapted, these host-derived specimens will be more representative of modern, medically-relevant pathogens. 

Questions of clinical translation and applicability of in vitro models arise not only with the choice of strain, but also with the methodology applied. The past several years have witnessed a rapid expansion in the laboratory characterization of Bb persister cells and pleomorphic forms, accompanied by the speculation that they represent missing links between microbiology and longstanding illness. The challenge now lies in reconciling these artificially-induced forms with persistent Bb that have been identified in model organisms and human cases, to determine whether the lab versions are reasonable approximations of the natural form, and therefore, whether they represent a basis for understanding treatment-refractory *Borrelia*. Stationary phase cultures are a common source of persistent Bb in the laboratory, but it remains to be seen whether they truly resemble pathogens under siege in a complex host environment.

Much of this in vitro work is oriented toward identifying effective therapeutic agents for use in recalcitrant disease, based on the distinct findings that a considerable fraction of patients report ongoing illness after the recommended duration of antibiotics (e.g., PTLDS), and that residual *Borrelia* have been observed in some antibiotic-treated animals and humans. Whether there is a need for optimized first-line drugs that more effectively and immediately limit disease progression and do not predispose patients to complications may be considered. Meanwhile, anti-persister cocktails that are being explored in vitro may predict the clinical adoption of increasingly aggressive, broad-spectrum antimicrobial chemotherapy for the treatment of longstanding disease. These strategies are opposite to those of other fields like oncology, in which emphasis is placed on developing more targeted therapeutics with limited collateral damage. Generating a more holistic understanding of the pathogens and their interface with the human host through multidisciplinary investigations, like the one depicted in Figure 6, is anticipated to yield data that can improve both diagnostic and treatment specificity.

### 6.3. Interdisciplinary Solutions

Across the body of literature, it is also apparent that the field has suffered from considerable polarization and siloed practices of medicine, research, and advocacy, to the detriment of those affected by Lyme disease. Perhaps not surprisingly, medical application has generally outpaced fundamental mechanistic microbiology, yet the discipline has reached an impasse of sorts that can only truly be resolved by harmonizing efforts. Reconciling existing knowledge and generating novel insight are both critical in this space, but meaningful progress at the societal level will not be achieved until a variety of solution-oriented stakeholders are strategically engaged (Wilson et al., 2019, in revision). 

## Figures and Tables

**Table 1:** United States Centre for Disease Control and Prevention (CDC) Lyme disease surveillance case definitions by disease stage.

Disease Classification		CDC Surveillance Definition
**Early**	Confirmed	EM + exposure in high incidence region (if exposure occurs in low incidence state or is unknown, laboratory evidence * is required)
	Probable	Physician diagnosed Lyme disease with laboratory evidence *
	Suspected	Case of EM with no known exposure & no laboratory evidence *
**Late**		Objective signs of specific organ damage + concurrent laboratory evidence *

* Laboratory evidence as defined by CDC (U.S.A.) refers to one of the following: (1) a positive culture for *B. burgdorferi,* (2) a positive two-tiered serology test consisting of a positive or equivocal ELISA first tier and a positive IgG (or IgM if within 30 days), or (3) a positive single-tier IgG Western blot (recommended for surveillance purposes only, not for patient diagnosis).

**Table 2:** Disease stage definitions (clinical findings and microbiological correlates).

Stage	Description	Microbiology	References
**Early Localized Lyme Disease** *7–14 days after inoculation*	- Can present with a single EM lesion (~70% of patients) - Can present with mild flu-like symptoms (fatigue, headache, myalgias, arthralgias and fever)	Localized *Borrelia* infection	[64,65,66]
**Early Disseminated Lyme Disease** *Days to months*	- Can present as multiple EM lesions, acute neurologic disease, Lyme carditis, borrelial lymphocytoma, and/or systemic symptoms	*Borrelia* enters the bloodstream and disseminates systemically	[64,65,66,67]
**Late Disseminated Lyme Disease** *Months to years (untreated)*	- Intermittent or ongoing objective signs of specific organ damage to joints (arthritis), heart (Lyme carditis), nervous system (encephalitis, polyneuropathy), and/or skin (acrodermatitis chronica atrophicans and lymphocytoma)	Ongoing *Borrelia* infection of tertiary organ sites	[66,68,69]
**Post-Treatment Lyme Disease Syndrome (PTLDS)** (previously referred to as Post Lyme Disease Syndrome/PLDS)	- Previous objective evidence of infection - Treated with antibiotics followed by resolution of objective signs - Onset of subjective symptoms (fatigue, widespread musculoskeletal pain and cognitive difficulties—see below for more information) within 6 months of treatment, that persists for at least 6 months	*Borrelia* infection that has been treated with antibiotics. There are several hypotheses for the cause of ongoing symptoms	[54,66]
**Chronic Lyme Disease (CLD)**	- Ongoing symptoms consistent with Lyme disease (fatigue, arthralgias, myalgias, nervous system dysfunction) not meeting strict definitions of another category	Topic of controversy	[67,70]

**Table 3:** Summarized in-vitro evidence of host–*Borrelia* interactions and methods of analysis.

Host Material	Host–*Borrelia* Interaction	Methods of Analysis	Citations
Chondrocytes	Adherence	Protein interaction analyses	(Behera et al., 2008) [206]
Collagen Matrix	Adherence and Sequestered	Adherence assay, BacLight	(Zambrano et al., 2004) [203]
Decorin (ECM proteoglycan)	Adherence	ELISA, IF, Protein interaction analyses	(Guo et al., 1995) (Guo et al., 1998) [207,208]
Dendritic Cells	Internalized	Darkfield microscopy, CLSM, BacLight	(Suhonen et al., 2003) [209]
Endothelial Cells	Adherence and Internalized	IF	(Ma et al., 1991) (Wu et al., 2011) [210,211]
Fibroblasts	Internalized	IF, Antibiotic Challenge & Re-Growth, RT-PCR	(Wu et al., 2011) (Klempner et al., 1993) [211,212]
Fibrocytes	Held in membrane invaginations	Light and electron microscopy	(Grab et al., 1999) [213]
Fibronectin Matrix	Adherence	Darkfield microscopy and ELISA	(Brissette et al., 2009) [214]
ECM Glycosaminoglycans	Adherence	Protein interaction analyses and liquid scintillation, IF	(Lin et al., 2015) (Parveen et al., 2003) [215,216]
Laminin Matrix	Adherence	ELISA	(Brissette et al., 2009) [214]
Lymphocytes	Internalized	Light microscopy, TEM, SEM	(Dorward et al., 1997) [180]
Macrophages	Internalized	CLSM, Re-Culture	(Montgomery et al., 1996) [217]
Monocytes	Internalized	Cytokine response analyses, CLSM	(Salazar et al., 2009) [218]
Neuronal and Glial Cells	Internalized	IF, Antibiotic Challenge	(Livengood et al., 2006) (Williams et al., 2018) [219,220]
Platelets	Adherence	IF	(Coburn et al., 1994) (Coburn et al., 1993) [221,222]
Synovial Cells	Internalized	CLSM and TEM, IHC on 3D model, Antibiotic challenge	(Girschick et al., 1996) (Franz et al., 2001) [223,224]

**Table 4:** Summary of distinct pleomorphic forms of *Borrelia* spp. (individual and community phenotypes) with speculated involvement in Lyme disease.

Term	Defining Features	Documentation	Verdict
Cyst	Encasement of a vegetative spirochete within a defined outer membrane.	(Miklossy et al., 2008) [313]	Encasement within an outer membrane reported to occur. Defining features of outer envelope/membrane unclear whether it meets criteria for cyst.
Cell Wall Deficient (CWD) Form/L-form/Spheroplast/Protoplast	Absence, or partial absence, of the cell wall. Absence of murein.	(Mursic et al., 1996) [316]	Does not seem to be the case as studies show the presence of peptidoglycan in the outer envelope/membrane of the round body.
Biofilm	Structurally rearranging aggregates within a matrix composed of extracellular polymeric substances (EPS) that attaches to biotic and abiotic surfaces.	(Sapi et al., 2012) [325]	Clusters and aggregates can be seen. Growth on collagen, fibronectin, and agarose confirmed. Alginate, calcium, and eDNA present in matrix.
Microcolony/Aggregate	Assemblies of spirochetes that are either adherent to a solid surface or free-floating within liquid. May include bundles or radial clusters.	(Feng et al., 2019) [326]	Evident in culture. Evidence in animal and clinical models unclear.
Spherule/Coccoid Form/Round Body	Spirochetes that have adopted a rounded, spherical morphology while retaining an intact outer membrane.	(Meriläinen et al., 2015) [314]	Evident in culture. PI staining indicates that the outer envelope/membrane is not intact.

## Abbreviations

AA: arachidonic acid; AI-2: autoinducer-2; Bb: *Borrelia burgdorferi*; BSA: bovine serum albumin; CDC: Centers for Disease Control and Prevention; CLD: Chronic Lyme Disease; CLSM: conventional light microscopy; CRASP: complement regulator-acquiring surface protein; CRP: C-reactive protein; CWD: cell wall deficient; CYTP: cytochrome P450; ECM: extracellular matrix; ELISA: enzyme-linked immunosorbent assay; EPA: eicosapentaenoic acid; EPS: extracellular polymeric substances; GC: germinal centres; HLA: human leukocyte antigen; ICs: immune complexes; IDSA: Infectious Diseases Society of America; IF: immunofluorescence microscopy; IHC: immunohistochemistry; ILADS: International Lyme and Associated Diseases Society; JHR: Jarisch-Herxheimer Reaction; LD: Lyme disease; LOX: lipoxygenase; MBC: minimum bactericidal concentration; MIC: minimum inhibitory concentration; MLST: Multilocus sequence typing; NSAIDs: non-steroid anti-inflammatory drugs; Osp: Outer surface protein; PCR: polymerase chain reaction; PGs: prostaglandins; PTLDS: post-treatment Lyme Disease Syndrome; PUFA: poly-unsaturated fatty acids; RB: round body; RCT: randomized controlled trial; RST: Ribosomal spacer typing; SEM: scanning electron microscopy; TEM: transmission electron microscopy; TRLA: treatment-refractory Lyme arthritis.

## Appendix A

**Table A1:** In vitro pleomorphic induction strategies. To compile the following information, we conducted a series of searches within PubMed Central, Primo, and Google Scholar using the search terms “*Borrelia*,” “Cyst,” “Cell Wall Deficient,” “L-form,” “Biofilm,” “Microcolony,” “Aggregate,” “Spherule,” “Coccoid,” “Round Body,” “Granule,” “Pleomorphic,” and “Lyme”.

Induction Method	Species/Strain	Conversion Kinetics	Morphology	Viability	Reference
**Distilled Water**	*B. burgdorferi* ACA-1	> 95% converted within 1 min 100% RBs by 4 h; all globular. Aggregates of round bodies formed at 1 week.	Round; spirochete whirled into itself after expansion of outer membrane.	Assessed viability by reversion and propagation of motile spirochetes from round bodies. Also confirmed the presence of RNA in round bodies using an acridine orange stain.	[328]
	*B. burgdorferi* B31 and ADB1	Majority converted within 5 min.	Tangled into a general spherical shape approximately 3–5 μm in diameter.	Viability was indicated by reversion into helical spirochetes following immersion in BSK-II. Could not exclude possibility of unconverted spirochetes contributing to the observed “reversion”.	[313]
	*B. burgdorferi* B31 and *FlaB* mutant (MC-1)	> 95% converted within the first 2–3 h.	Globular (approximately 1 μm in diameter) with the end of the cylinder protruding outwards.	Mobile spirochetes were recovered after incubation in BSK-II from 4-day old round bodies. RNA was also isolated even after 16 days in distilled water.	[329]
	*B. burgdorferi* B31	85% converted by 10 min.	Spherical with a mean diameter of 2.8 μm. TEMs depicted intact inner and outer membranes.	10 min and 2 h round bodies reverted back to helical spirochetes and reached a concentration of 4.0 × 10^7^/mL after incubation in BSK-II for 6 and 8 days respectively.	[314]
**Cerebral Spinal Fluid**	*B. burgdorferi* ACA-1	100% conversion within 24 h (37C incubation). Cyst formation was different depending on concentration of spinal fluid protein (higher = slower conversion).	Spherical, approximately 2 μm in diameter.	Reversion to motile spirochetes of logarithmic phase density when reintroduced into BSK medium.	[319]
**RPMI-1640**	*B. burgdorferi* B31 (high-passage) and T15 (low-passage)	90% converted into “cysts” by 48 h.	Twisted and knotted into a rounded form with an outer envelope. Approximately 2–3 μm in diameter.	Immediate reversion to non-motile, intact spirochetes upon the addition of rabbit serum or BSK. Cysts did not open when 20% sucrose was added to the culture. Cells began to regain motility 12–15 h after emerging from the cysts.	[353]
	*B. burgdorferi* B31	17% converted into round bodies by day 4.	Spherical with folded spirochetal cylinder within a membrane. TEMs depict intact inner and outer membranes. Approximately 2.8 μm in diameter.	Did not specify.	[314]
	*B. burgdorferi* 297	Quantitative data not provided. Δ*relB* mutants converted faster than their wild type counterparts in the RPMI medium.	Spherical with either smooth surfaces or rough/blebbing surface. Spirochete appears to fold and curl within a round membrane with part of cylinder protruding out. Approximately 1.2–2 μm in diameter.	A small proportion of the round bodies did not stain with propidium iodide, indicating intact membranes and subsequent viability.	[225]
**Serum free BSKII (BSKII–S) or BSK-H (-rabbit serum)**	*B. burgdorferi* ACA-1	By one week, round “cystic” structures were seen at an unspecified proportion. By six weeks, only cystic structures were seen.	A combination of round and irregular shapes with a mean diameter ranging from 0.5–2.0 µm.	When re-introduced to BSK-H with rabbit serum, mobile spirochetes were seen after six weeks. Likewise, re-introduction of filtrate (un-transformed spirochetes) to regular BSK-H failed to yield mobile spirochetes or growth for at least three months	[354]
	*B. burgdorferi* B31	> 95% converted in 7–10 days.	Morphologically similar to the distilled water induction within the same experiment.	Did not specify.	[329]
	*B. burgdorferi* B31	No different than control.	NA	NA	[314]
**Oxidative Stress**	*B. garinii* BITS	6% converted over 24 h (in MEM and H_2_O_2_). Rapid conversion of majority within 10 min (in H_2_O and H_2_O_2_).	Round expansion of outer membrane with folding of cylinder within. Size undetermined/not specified.	Reversion back to helical spirochetes took over 2 months.	[321]
**Changes in pH**	*B. garinii* BITS	Conversion of small fraction at pH 2 and 11 after 30 min.	Not specified.	Not specified.	[321]
	*B. burgdorferi* B31 and ADB1	Not specified.	Not specified	Not specified.	[313]
**Changes in Temperature**	*B. garinii* BITS	“Cystic” forms increase as temperature increases when incubated in MEM.	Not specified.	Reversion to helical spirochetes within 30 days of incubation under regular growth conditions.	[321]
	*B. burgdorferi* B31 and ADB1	Not specified.	Not specified.	Not specified.	[313]
**Human Serum**	*B. burgdorferi* B31	22% converted to round bodies after 4 days.	Spherical with a mean diameter of 2.8 μm. TEMs depicted intact inner and outer membranes.	Not specified.	[314]
**Antibiotics**	*B. burgdorferi* B31	96% converted into round bodies following a 3 day incubation in 50 μg/mL of amoxicillin.	Not specified.	Reversion to normal spirochetes within 5 days of subculture in BSK-H.	[334]
	*B. burgdorferi* B31, H1, and H8	“Spherical bodies” were present after 18 h in cultures with penicillin, ceftriaxone, and doxycycline.	“Spherical bodies” were described as being 0.8–1.4 μm in diameter. TEM depicts an electron dense sphere surrounded by an outer membrane.	Subculture from all samples failed to yield morphologically typical *Borrelia* by two weeks.	[333]
**Eukaryotic Cell Co-Culture/Infection**	*B. burgdorferi* B31 and ADB1	1 week of incubation with chicken primary sympathetic neurons and rat astrocytes seem to yield “ring shaped cystic forms” that can aggregate.	The average “ring shaped cystic form” appears to be anywhere from 1–2 μm in diameter.	Not specified.	[313]
